# Tunable redox hopping charge transport and electrochromism in multivariate MOFs: effects of substitution patterns and number of sulfonic acid groups

**DOI:** 10.1039/d6sc02965e

**Published:** 2026-06-04

**Authors:** Sumanta Basak, Laura Prince, Zhengyu Du, Amanda J. Morris

**Affiliations:** a Department of Chemistry, Virginia Polytechnic Institute and State University Blacksburg Virginia 24061 USA ajmorris@vt.edu; b Macromolecules Innovation Institute, Virginia Polytechnic Institute and State University Blacksburg Virginia 24061 USA

## Abstract

Controlling fundamental electron and ion transport in porous materials is a critical challenge because it dictates the performance, speed, and durability of devices ranging from batteries to high-speed sensors. Electrochromic devices change color under applied voltage, but fast and reversible switching requires efficient electron transfer and ion motion. Metal–organic frameworks (MOFs) offer tunable electron and ion transport, but charge mobility is often limited by slow ion diffusion. Our prior work revealed that sulfonation enables exceptionally fast charge transport and electrochromism. However, developing strategies that allow simultaneous and molecular-level control of electron and ion transport within a single framework remains a central challenge. Here, we demonstrate that systematic sulfonation of MOF linkers provides a direct molecular handle to regulate charge transport in ruthenium-loaded UiO-67 MOFs. By varying the number and position of –SO_3_H groups on MOF linkers, we isolate sulfonation density and spatial arrangement as independent variables governing charge transport. The 3,3′-SO_3_H framework exhibited faster charge transport than the 2,2′-SO_3_H analogue, underscoring the significance of the sulfonation position. Increasing the number of sulfonic acid groups further enhanced performance, with the tetra-substituted 3,3′,5,5′-SO_3_H variant showing the fastest coloration (1.09 ± 0.05 s) and bleaching (1.5 ± 0.2 s) times and an apparent diffusion coefficient (*D*_app_) of (5 ± 3)×10^−6^ cm^2^ s^−1^ and a coloration efficiency of (641 ± 12) cm^2^ C^−1^. Raman spectroscopy reveals a mechanism of strong sulfonate–cation interactions, in which sulfonation promotes ion-pair dissociation and facilitates ion motion. Together, these results establish linker sulfonation as a general strategy for controlling both ion-electron transport in MOFs, with enhanced electrochromic performance emerging as a direct manifestation of optimized charge transport.

## Abstract 2

Systematic sulfonation of UiO-67 linkers tunes ion transport and electrochromic switching in Ru-loaded MOFs.

## Graphical abstract

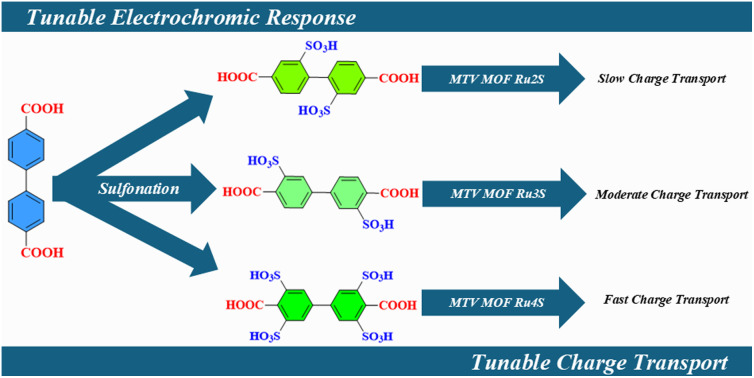

## 1. Introduction

MOFs, a class of porous coordination polymers (PCPs), are composed of metal-containing nodes (secondary building units, or SBUs) and organic linkers.^1^ Because of their structural and functional flexibility, MOFs have quickly become one of the most dynamic topics in chemistry. MOFs are known for their extremely high surface areas, customizable pore sizes, and adjustable internal surfaces. These desirable properties make them highly promising for applications in drug delivery,^2^ storage media for gases,^3^ chemical sensing^4^ and catalysis.^5^ In many of these applications, especially those involving ion or molecule transport, such as in electrochromic devices, electrocatalysis, redox flow batteries, and proton exchange membranes, the rate of diffusion of charge-carrying species through the MOF becomes a key performance-limiting factor.^6^ The apparent diffusion coefficient (*D*_app_) is a crucial metric for describing total charge transport in the study of charge transfer in MOFs. *D*_app_ takes into consideration two interrelated processes: counterion diffusion to balance the resultant charge and electron hopping between redox centers (self-exchange). As such, understanding and controlling diffusion coefficients within MOF structures is crucial for optimizing material performance and enabling their practical deployment in device architectures. The production of MOF thin films on conducting substrates has made it easier to characterize these systems electrochemically and increased their applicability in a greater range of applications.^7^ The Morris group has previously examined the charge transfer behavior of MOFs using the Scholz model which describes charge transport within a microporous particle situated on an electrode surface.^8,9^ Application of the Scholz framework revealed that the primary limitation in charge transport arises from the diffusion of counterbalancing ions.^10^ Typically, MOFs display redox charge transfer with *D*_app_ in the range of (2 ± 1) × 10^−10^ cm^2^ s^−1^.^11^

While MOFs were once thought to be electrically insulating, recent work has shown that efficient charge transport is achievable through careful selection of redox-active ligands and metal nodes.^12^ Additionally, post-synthetic modifications, particularly using coordinatively unsaturated metal sites, allow for the incorporation of electroactive species, enhancing the electronic properties of the frameworks.^13^ Charge transfer in MOFs is influenced by a complex interplay of pore structure, framework flexibility, guest–host interactions, and functional group chemistry.^14^ While earlier studies have primarily focused on structural control, such as pore size, connectivity, and topology, to influence diffusion, chemical functionalization of MOF linkers has emerged as a powerful but underexplored route to modulate transport properties.^15^

Electrochromic materials, which reversibly modulate their optical properties upon redox switching, are central to technologies such as smart windows, low-power displays, and optical sensors.^16^ While transition-metal oxides remain the dominant class of electrochromics, their switching kinetics are often limited by ion intercalation processes that lead to sluggish coloration and/or bleaching responses. The primary limitation of these materials lies in their constrained capacity for color tuning, coloration efficiency, and chromatic saturation.^17,18^ In contrast, conducting polymers and small organic molecules exhibit pronounced chromatic diversity; however, they frequently suffer from inadequate long-term stability. Furthermore, both metal oxides and conducting polymers are restricted by inherently slow switching kinetics. Although several of these materials demonstrate considerable potential and some have already reached commercial deployment, the development of energy-efficient systems with rapid response characteristics remains a critical research priority. Electrochromic MOFs reported to date largely rely on intrinsically redox-active organic linkers (*e.g.*, porphyrins or rylenediimide derivatives), where charge transport and optical modulation are inherently coupled to the chromophore's electronic structure. Studies by the Dincă and Ott groups demonstrated multicolor electrochromism and high coloration efficiency in diimide-based MOFs; however, variations in linker structure, electronic coupling, and framework orientation convolute ion transport and redox hopping, hindering isolation of a single structure–transport relationship.^19,20^ Our previous work demonstrated that introducing sulfonic acid groups (–SO_3_H) into the framework of Ru(bpy)-UiO-67 using a multivariate (MTV) approach significantly enhanced charge transport for the very first time. Specifically, substitution at the 3,3′ positions of biphenyl dicarboxylate linkers promoted complete redox conversion and yielded one of the fastest reported *D*_app_ values for a redox-hopping MOF, enabling rapid and reversible electrochromic switching.^21^ However, the structure–function relationship governing the enhancement, specifically how the substitution pattern and number of sulfonic acid groups affect diffusion and electrochromic response, remained unresolved. By varying both the numbers of –SO_3_H groups and how they are substituted systematically, we can isolate the effects that counter-ion transport and local electrostatic interactions have on the mechanisms of redox hopping, which in turn provide valuable mechanistic information about how ion-coupled electron transfer occurs in electrochromic porous materials. A more systematic understanding of these parameters is essential for designing MOFs with tailored ionic transport properties, especially for electrochemically active systems, particularly where rapid ion movement through the substrate is required. Expanding on that research, the present work advances the field by systematically disentangling how the number and substitution pattern of –SO_3_H groups regulate redox hopping and electrochromic kinetics in a multivariate MOF platform. The study isolates sulfonation density as a mechanistic variable controlling apparent diffusion coefficients and switching dynamics. The approach transforms sulfonation from a qualitative design motif into a quantitative handle for tuning charge transport in electrochromic MOFs, providing fundamental insight that is difficult to access in systems where the redox unit and transport pathway are intrinsically linked.

Building upon these findings, herein, we investigate the structure–function relationships of sulfonation patterning in UiO-67 MOF films with electrochromic Ru(tpy)(dcbpy) (tpy = 2,2'; 6′,2″-terpyridine; dcbpy = 5,5′-dicarboxylic acid-2,2′-bipyridine) (RuTPY) linkers incorporated along with the substituted biphenyl dicarboxylic acid (BPDC) in an MTV approach. MTV MOFs enable the incorporation of multiple functional groups in well-defined spatial arrangements, resulting in enhanced activity that exceeds the sum of the individual components.^22^

**Fig. 1:**
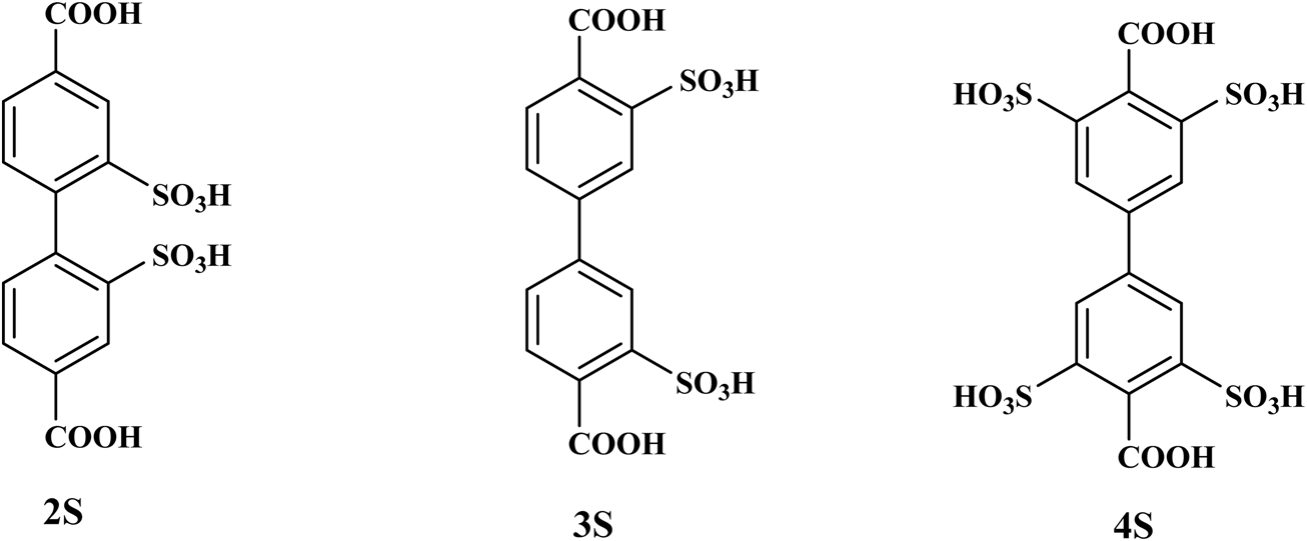
Chemical structures of the sulfonated biphenyl linkers used to construct the multivariate MOFs.

Specifically, we compare frameworks incorporating –SO_3_H substituents at the 2,2′ positions, the 3,3′ positions, and simultaneously at two sites (3,3′,5,5′ functionalization), thereby varying the number (two *vs.* four) and spatial arrangement of acidic groups within the framework. For clarity, RuTPY-UiO-67 functionalized with sulfonic acid groups at the 3,3′ positions of the biphenyl dicarboxylate linker is hereafter referred to as Ru3S. The analogous 2,2′-substituted and 3,3′,5,5′-substituted frameworks are referred to as Ru2S and Ru4S, respectively (Fig. 1). The native RuTPY-UiO-67 MOF films showed slow charge transfer rates with a *D*_app_ of (2 ± 2) × 10^−9^ cm^2^ s^−1^ while converting only 57% of the redox-active sites. Upon incorporation of sulfonate groups, charge transport improved dramatically: tetra sulfonated MOFs achieved complete (∼100%) redox conversion, and their *D*_app_ values increased by orders of magnitude. Notably, the tetra-substituted Ru4S variant exhibited the highest charge transport rate, with a *D*_app_ of (5 ± 3) × 10^−6^ cm^2^ s^−1^, representing the fastest redox hopping rate reported for this class of materials to date. The enhanced ion mobility afforded by sulfonation enabled rapid and reversible electrochromic switching, underscoring the critical role of linker functionalization in dictating charge transport properties.

## 2. Results and discussion

### 2.1 Synthesis and characterization of MTV-MOFs

A series of MTV MOFs were prepared by incorporating sulfonated BPDC linkers into a standard UiO-67 synthesis. In a typical procedure, zirconium chloride (58.25 mg, 0.25 mmol), sulfonated BPDC (50.8 mg, 0.21 mmol), and [Ru(tpy)(dcbpy)Cl]PF_6_ (32.6 mg, 0.043 mmol) were combined in a 6-dram vial containing DMF (10 mL) and glacial acetic acid. A cleaned FTO slide was placed in the vial with its conductive side down. The sealed vial is heated at 120 °C for 24 h, yielding thin films on the FTO. Following synthesis, the films were removed, washed thoroughly with DMF, and then soaked in fresh water for 24 h to exchange the chloride ligand and generate the chromophore [Ru(tpy)(dcbpy)(H_2_O)]PF_6_.

**Fig. 2:**
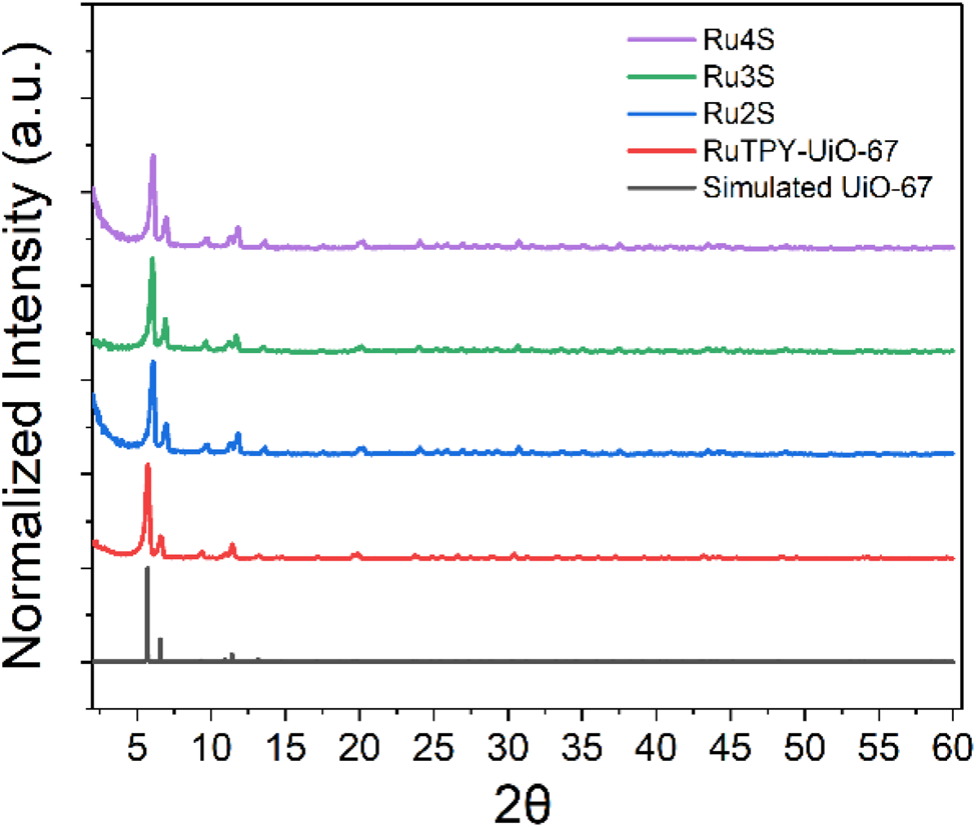
PXRD patterns of RuTPY-UiO-67 (red), Ru2S (blue), Ru3S (green), and Ru4S (violet) thin films as compared to simulated UiO-67 (CCDC 1021002), collected using Cu K_α_ radiation (*λ* = 0.1541 nm).

**Fig. 3:**
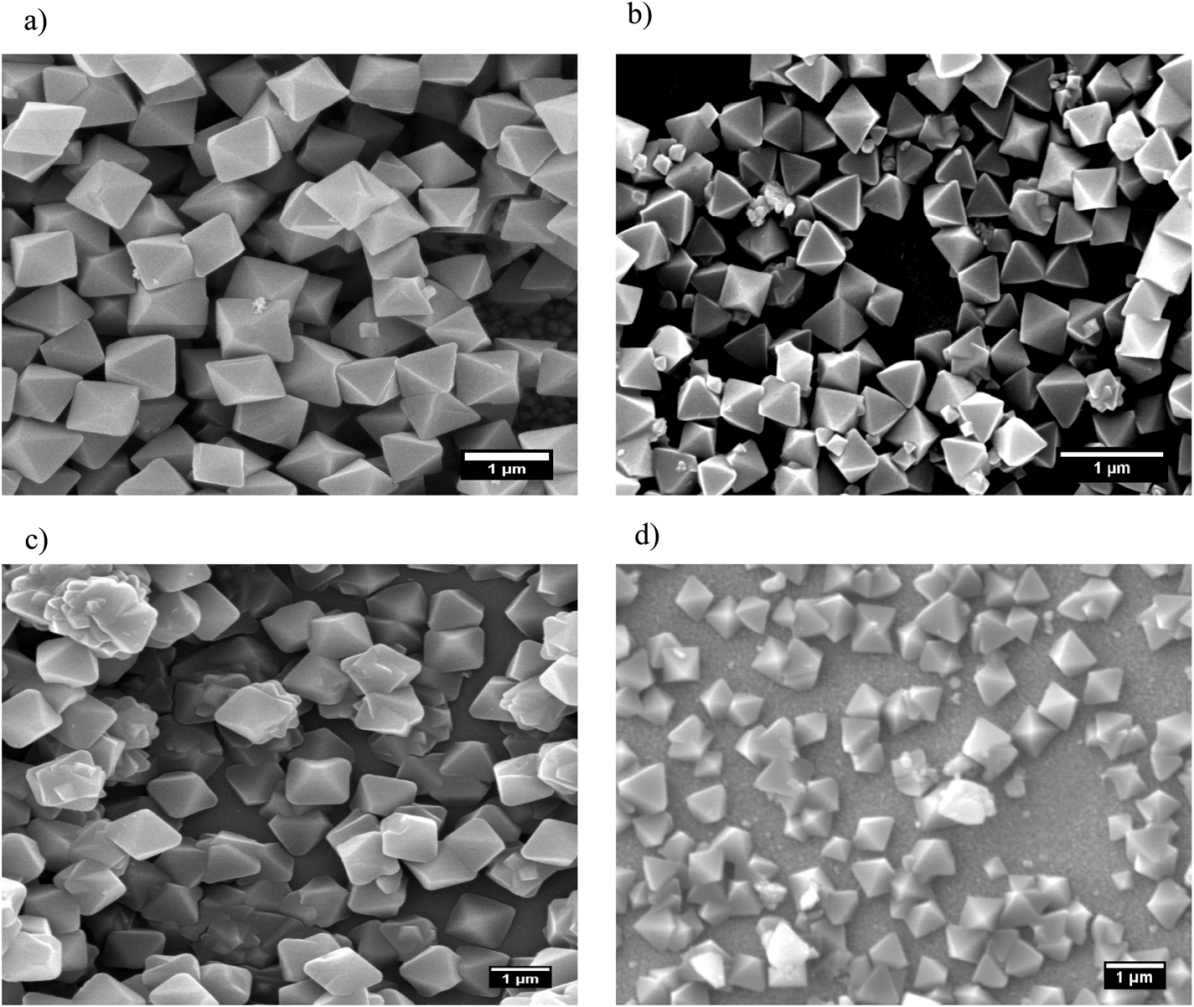
SEM images of (a) RuTPY-UiO-67, (b) Ru2S, (c) Ru3S, and (d) Ru4S MOF thin films.

The crystallinity and morphology of the synthesized thin films were evaluated using powder X-ray diffraction (PXRD) and scanning electron microscopy (SEM). The PXRD patterns of all MOF samples exhibit a sharp reflection at 2*θ* ≈ 5.5°, accompanied by a weaker peak at 6.4°, in excellent agreement with the simulated diffraction pattern of UiO-67 (Fig. 2), confirming the formation of the expected framework. SEM analysis reveals well-defined octahedral crystals for all samples, consistent with the typical morphology of UiO-type MOFs (Fig. 3a–d), indicating uniform growth and high-quality film formation. The relative stoichiometry between RuTPY chromophore units and sulfonated biphenyl linkers was quantified by digestion ^1^H NMR spectroscopy, a well-established method for determining linker and catalyst incorporation in mixed-linker MOFs.^22^ Integration of diagnostic proton resonances corresponding to the RuTPY chromophore and the sulfonated biphenyl linkers enables direct comparison of their relative ratios after complete framework digestion. Across all sulfonated MOF thin films, the RuTPY-to-sulfonated-linker ratios were determined to be ∼1 : 5 (Fig. S1). These ratios correspond to an average loading of approximately one RuTPY chromophore unit per Zr_6_ node, consistent with the designed framework stoichiometry. The thicknesses of all MOF thin films were determined from cross-sectional SEM images. For each sample, the film thickness was measured at a minimum of three distinct locations across the cross-section using ImageJ software, and the reported values represent the average thickness with the corresponding variability (Fig. S2). Specifically, the RuTPY-UiO-67 film exhibited thicknesses of 1.53, 2.24, and 2.29 µm, giving an average thickness of 2.02 ± 0.41 µm. The sulfonated films showed slightly increased but comparable thicknesses *e.g.* Ru2S (2.83, 2.94, and 3.02 µm; 2.93 ± 0.09 µm), Ru3S (1.83, 2.36, and 2.67 µm; 2.29 ± 0.42 µm), and Ru4S (2.90, 3.03, and 3.14 µm; 3.03 ± 0.12 µm). Overall, all films fall within a thickness range of approximately 2–3 µm.

### 2.2 Electrochromic response

**Fig. 4:**
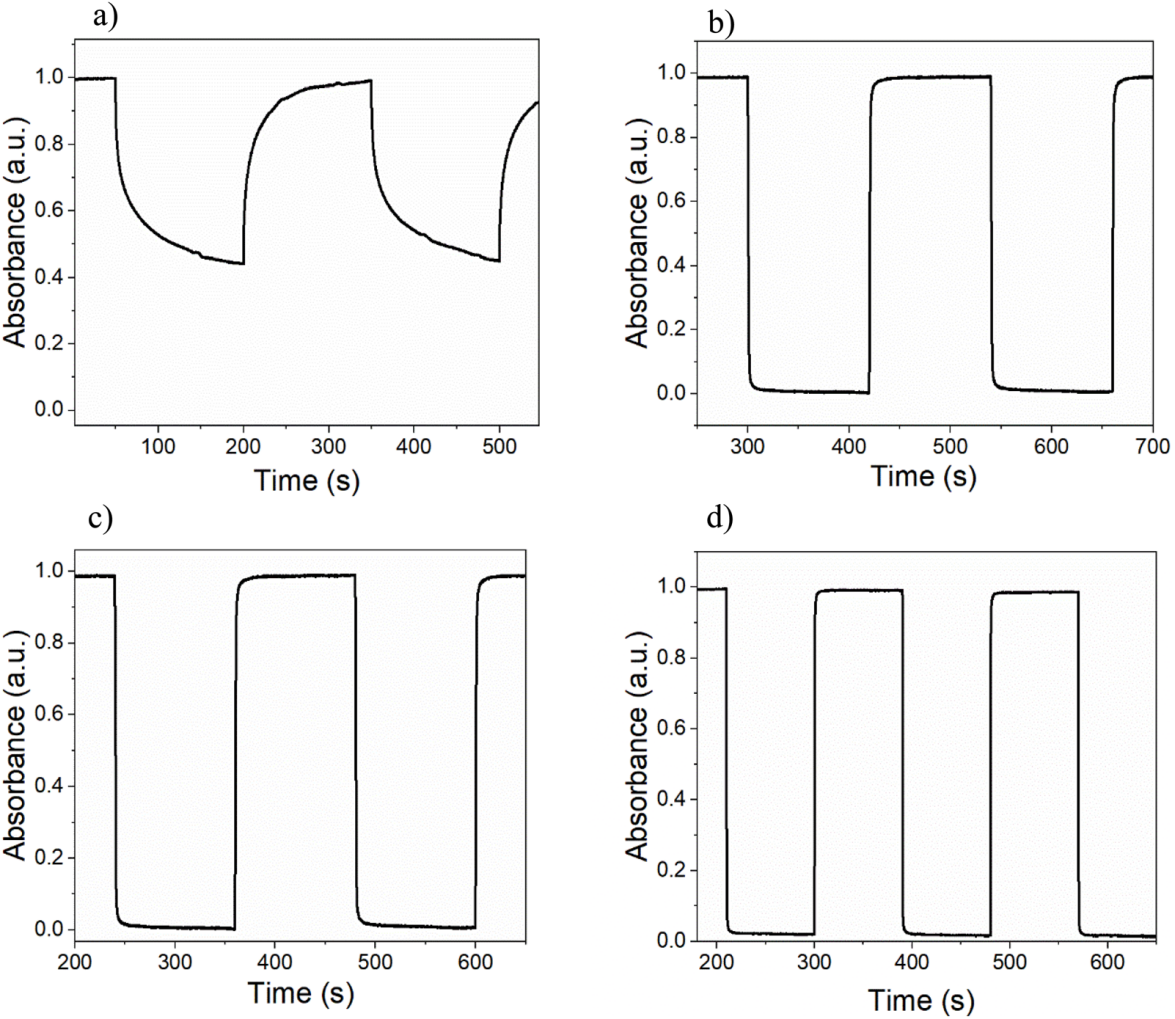
Time-dependent absorbance profiles of (a) RuTPY-UiO-67, (b) Ru2S, (c) Ru3S, and (d) Ru4S, normalized to unity at the Ru^2+^ state and zero at the expected Ru^3+^ state.

The electrochromic response kinetics of the MOF films were investigated by monitoring the time-dependent change in absorbance during successive redox cycles. Native RuTPY-UiO-67 and its sulfonated derivatives (Ru2S, Ru3S, and Ru4S) were evaluated under identical spectroelectrochemical conditions (Fig. 4). Specifically, the absorbance at 520 nm was recorded while stepping the potential to 1800 mV *vs.* NHE (overpotential ∼400 mV), followed by relaxation at the open-circuit potential (Fig. S3). Because Ru^3+^ exhibits ∼85% lower molar absorptivity than Ru^2+^ at this wavelength, an ∼85% decrease in absorbance corresponds to full oxidation and redox conversion.^23^ The tetra sulfonated films exhibit ∼85% decrease in absorbance, indicating ∼100% redox conversion, whereas the parent RuTPY–UiO-67 film shows only ∼57% absorbance loss, consistent with incomplete conversion. These measurements enabled direct comparison of switching dynamics and reversibility as a function of sulfonation pattern.

For Ru2S, the oxidation (bleaching) and reduction (coloration) processes occur at nearly identical rates of (0.19 ± 0.01) s^−1^ and (0.23 ± 0.04) s^−1^, corresponding to time constants of approximately (5.26 ± 0.28) s and (4.35 ± 0.78) s. For Ru3S, these processes occur at rates of (0.29 ± 0.02) s^−1^ and (0.38 ± 0.03) s^−1^, corresponding to time constants of approximately (3.45 ± 0.24) s and (2.63 ± 0.21) s. The close match in rates indicates a highly reversible electrochromic response with minimal hysteresis. The relatively fast switching underscores efficient electron transfer and ion mobility within the framework, which can be attributed to the presence of sulfonic acid functionalities that facilitate charge transport. From the comparison between Ru2S and Ru3S, we found that when the sulfonate groups are positioned close to each other (2,2′-positions), charge transfer is less efficient than when they are located farther apart (3,3′-positions). We hypothesize that when the sulfonate groups are too closely spaced, electrostatic agglomeration between the bulky SO_3_^−^ groups and the cations of the supporting electrolyte may lead to partial charge neutralization, thereby attenuating charge transport. To gain molecular-level insight into the role of sulfonate spacing, electrostatic potential (ESP) maps of the 2,2′- and 3,3′-disulfonated biphenyl linkers were computed using density functional theory (Fig. S4). The 2,2′-substituted linker exhibits closely spaced negative electrostatic potential between the two sulfonate groups, forming a localized region of high charge density. In contrast, the 3,3′-substituted analogue shows spatially separated regions of negative potential, indicating more independent charge sites. This distinction suggests that the closely spaced sulfonates in the 2,2′ configuration promote multidentate interactions with cations, favoring the formation of contact ion pairs or aggregates that effectively screen the charge and hinder ion mobility. Conversely, an increased number of sulfonate groups is expected to enhance the dissociation of ion pairs in solution, resulting in improved ion diffusion. These aspects are discussed in detail in a later section. In the case of Ru4S, both oxidation and reduction rates also show strong symmetry, measured at (0.67 ± 0.10) s^−1^ and (0.92 ± 0.04) s^−1^, translating to response times of about (1.5 ± 0.2) s and (1.09 ± 0.05) s. These values point to even faster dynamics compared to both Ru2S and Ru3S. The clear acceleration indicates that a higher density of ion-conducting groups directly promotes charge mobility and switching efficiency. By contrast, the native, non-sulfonated RuTPY-UiO-67 framework switches far more slowly. Its oxidation and reduction proceed at (0.05 ± 0.03) s^−1^ and (0.02 ± 0.04) s^−1^, respectively, which translates to characteristic times an order of magnitude slower than those observed in the sulfonated analogues. Although the forward and backward processes remain fairly balanced, the absolute response is limited, highlighting that the absence of ion-conducting sulfonic groups imposes restrictions on both ion and electron transport, thereby constraining the electrochromic performance.

Optical contrast, defined as the change in optical transmittance between the colored and bleached states, is a key figure of merit for electrochromic materials.^24^ The optical contrast is calculated based on the transmittance measured in the UV-Vis region using the bare substrate as a baseline and then the optical contrast is determined at the MLCT band according to Δ*T* = (*T*_bleached_ − *T*_colored_) (Fig. S5). Using this approach, the parent RuTPY-UiO-67 film exhibits a moderate optical contrast of ∼57.1%, whereas systematic sulfonation leads to a pronounced enhancement, with contrasts of ∼78.2% (Ru2S), ∼82.4% (Ru3S), and ∼89.2% (Ru4S), placing the highly sulfonated films firmly in the high optical-contrast regime (>80%) (Table S1). Additionally, the optical contrasts of Ru3S and Ru4S are approaching the average optical contrasts typically found in high-performance electrochromic systems, along with very fast switching rates at low applied voltages. In addition to achieving large optical contrasts, efficient electrochromic materials must convert electrical input into optical modulation with minimal charge consumption. The durability of the synthesized MOF thin films was examined for 100 cycles (Fig. S6). To assess the charge-to-optical conversion efficiency and provide a comprehensive evaluation of electrochromic performance, we calculated the coloration efficiency (CE) of the Ru-based MOF films. Coloration efficiency (CE) is an important performance metric for electrochromic materials as it quantifies the change in optical density achieved per unit of injected charge. It indicates how well electrical energy has been converted into an optical response. The CE at a given wavelength (*λ*) was calculated according to eqn (1),^25,26^1
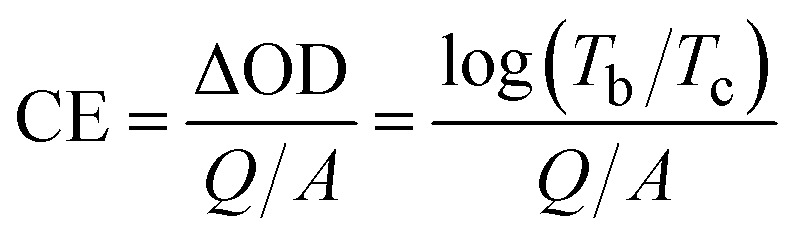
where ΔOD is the change in optical density at the selected wavelength, *Q* is the injected charge, *A* is the electrode area, and *T*_b_ and *T*_c_ are the transmittance values in the bleached and colored states, respectively. By following the above method, Ru-based MOF films show a high coloration efficiency of (225 ± 20) cm^2^ C^−1^ for Ru2S and (351 ± 23) cm^2^ C^−1^ for Ru3S, while that of Ru4S reaches (641 ± 12) cm^2^ C^−1^. The coloration efficiency increased systematically from Ru2S to Ru4S, consistent with the corresponding increase in optical contrast across the series. The progressive increase in CE with increasing sulfonation degree indicates that a larger fraction of the injected charge is effectively converted into optical modulation. The observed differences in coloration efficiency likely arise from a combination of factors, including variations in film thickness, active material loading, optical modulation, and charge transport properties within the MOF films. While the Ru loading slightly decreased from Ru2S to Ru4S, the more highly sulfonated frameworks exhibited faster electrochromic switching and high electrochemical accessibility of the Ru centers. The fact that Ru4S exhibits a CE of 641 cm^2^ C^−1^ is comparable to or exceeds inorganic oxides^27^ and most MOF-based electrochromic devices, suggesting that the sulfonated UiO-67 framework can achieve large optical density changes at lower levels of charge consumed during the process (Table S1).

When placed against traditional metal oxide electrochromic systems, all sulfonated frameworks clearly outperform in terms of response time, and they do so at much lower driving potentials. For example, amorphous WO_3_·2H_2_O films typically need 18 s for coloration/bleaching at 550 mV overpotential; sputtered WO_3_ films optimized for durability show 11 s and 5 s responses at 700 mV overpotential; and even the best-performing Ni–Co oxide thin films generally require 4 s at 300 mV overpotential.^28–30^ Vanadium-based counterparts such as VO_2_ and V_2_O_5_ can be much slower, reaching 35 s at 520 mV, while more advanced composite structures (*e.g.*, MoO_3_/V_2_O_5_ nanobilayers or Ti^4+^-doped V_2_O_5_) achieve intermediate switching in 6–8 s around 320 mV overpotential.^31,32^ In addition, our sulfonated MOFs surpass other electrochromic MOF systems (Tables S1–S3). To place our electrochromic MOF results in the broader context of framework materials, we also compared switching kinetics with representative covalent organic frameworks (COFs). Several COF systems exhibit relatively slow electrochromic responses, with coloration and bleaching times that are slower than or on the same order as conventional inorganic materials and significantly longer than those of our sulfonated MOFs (1.09 s coloration, 1.5 s bleaching). A triphenylamine-based COF thin film displayed ∼18.6 s coloration at 532 nm and ∼0.7 s bleaching under bias, indicating slower overall switching kinetics despite moderate bleaching speed.^33^ In another report, COF membranes exhibit ∼13.6 s coloration and ∼17.8 s bleaching, indicating that extended frameworks with hierarchical pores can still have slow charge transport.^34^ Multistate electrochromic COF films, used for safety indicators, show ∼4.9 s coloration and ∼10.6 s bleaching in the NIR region, and bipolar COF-GZU1 films exhibit switching times ranging from ∼7.8–14.5 s and ∼1.0–12.6 s across different wavelengths.^35,36^ Although there are instances of extremely rapid COFs,^37^ the average COF electrochromic responses are still slower than those found in our materials, underscoring the advantage of our approach in achieving rapid ion-coupled redox hopping and fast optical modulation across extended porous frameworks (Tables S1 and S2). The photographs of the MOF thin films in their respective colored and bleached states are shown in Fig. S7.

### 2.3 Measurement of diffusion coefficient (*D*_app_)

In redox-active MOFs, the *D*_app_ serves as a central parameter for describing charge transport. Unlike molecular systems in homogeneous solution, where diffusion reflects the translational motion of species, *D*_app_ in MOF thin films encompasses two interdependent contributions: (i) electron self-exchange (redox hopping) between immobilized redox centers, and (ii) the migration of counterions through the porous framework to maintain charge neutrality. Thus, *D*_app_ represents a collective descriptor of both electronic and ionic mobility within the film.^38^ A variety of electrochemical approaches can be employed to determine *D*_app_, including cyclic voltammetry (CV), chronoamperometry (CA), spectroelectrochemistry (SEC), and chronocoulometry (CC). These methods are attractive because they rely on widely available instrumentation such as potentiostats and UV-vis spectrophotometers, and they provide a direct link between electrochemical signals and transport phenomena. Nevertheless, the extracted values of *D*_app_ can differ significantly depending on the chosen technique, as each relies on distinct modelling assumptions and probes different aspects of the charge transport process.^39^

For example, CV is often used due to its simplicity and rapid data acquisition. In the method, *D*_app_ is inferred from the dependence of peak current on scan rate, assuming fast electron transfer kinetics, negligible ohmic drop, and semi-infinite linear diffusion. However, deviations from these assumptions, such as resistive films, low ionic conductivity, or background currents, can lead to underestimation of peak currents and thus artificially low *D*_app_ values. By contrast, CA evaluates the transient current response following a potential step. The Cottrell equation is typically applied under the assumption of semi-infinite diffusion, where the current decays proportionally to *t*^−1/2^. While the technique is less sensitive to ohmic drop after the initial milliseconds, early contributions from capacitive charging or film heterogeneity can complicate analysis. CC and Anson plot analysis further enables evaluation of *D*_app_ by integrating current over time, with the expectation of linearity between charge and the square root of time. While robust, deviations may arise in the presence of film roughness, porosity gradients, or residual background currents. Taken together, these methods highlight the complexity of determining *D*_app_ in MOF thin films.^39^ Importantly, *D*_app_ should be interpreted not as a pure diffusion coefficient in the classical sense, but rather as an effective transport parameter that integrates both electron hopping kinetics and ion mobility. Cross-validation between multiple electrochemical techniques and careful attention to experimental artifacts is therefore essential for obtaining reliable and meaningful values of *D*_app_ in MOFs. All *D*_app_*S* reported in this work represent the average values obtained from three independently prepared MOF thin films for each composition; the reported uncertainties correspond to the standard deviation across these independent samples.

#### 2.3.1 CV

**Fig. 5:**
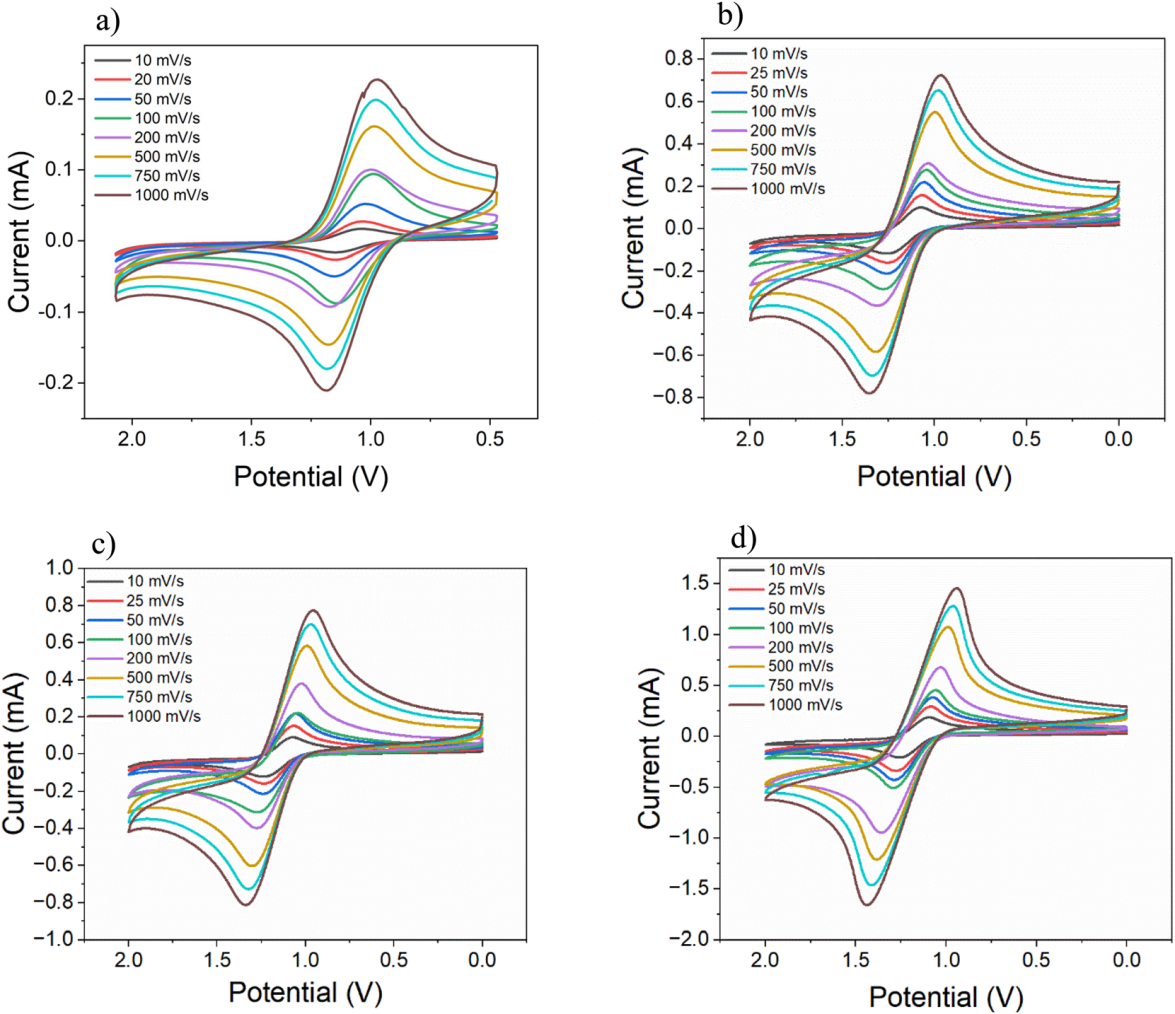
Cyclic voltammograms at varying scan rates for (a) RuTPY-UiO-67, (b) Ru2S, (c) Ru3S, and (d) Ru4S.

Scan rate-dependent CV provides a widely used approach for estimating the *D*_app_ of MOF thin films deposited on electrode surfaces. In the method, the relationship between the peak current response and the square root of the scan rate is analyzed, and the data are fitted to the Randles–Ševčík equation (eqn (2)). The formalism originates from the analytical solution of Fick's second law for a species undergoing diffusion under semi-infinite boundary conditions during a linear potential sweep, with the electrode interface behavior described by the Nernst equation.^40^ By applying these assumptions, the peak current becomes directly proportional to *D*_app_, allowing transport properties of immobilized redox centers within the MOF framework to be quantified.2
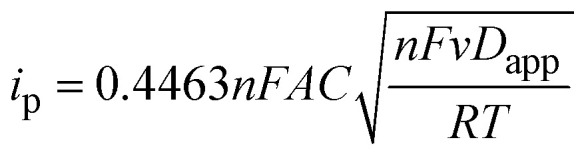
where *i*_p_ is peak current, *A* is electrode area, *F* is Faraday constant, *C* is concentration of the electroactive species, *D*_app_ is diffusion coefficient, *v* is scan rate, and *R* and *T* are the universal gas constant and absolute temperature. Fig. 5 shows scan rate-dependent cyclic voltammograms of RuTPY-UiO-67 and its sulfonated analogues.

The CVs of RuTPY-UiO-67 and its sulfonated derivatives (Ru2S, Ru3S, and Ru4S) display the expected Ru^2+/3+^ redox couples with clear trends in potential and reversibility as a function of sulfonation. All electrochemical potentials reported in this work are referenced to the normal hydrogen electrode (NHE). For the parent RuTPY-UiO-67, a reversible couple is observed at ∼1.07 V *vs.* NHE with a peak-to-peak separation of ∼100–120 mV and near-ideal peak current ratios. Introduction of two sulfonate groups in Ru2S and Ru3S results in anodic shifts of ∼100–150 mV relative to the native, with peak separations of ∼150–180 mV and slightly diminished reversibility, reflecting the electron-withdrawing nature of SO_3_H substituents and modestly slower electron-transfer kinetics. Ru4S, bearing four sulfonate groups, exhibits the most positive *E*_1/2_ (∼1.3–1.35 V) and the maximum peak separation, together with reduced peak current symmetry, indicating a more pronounced deviation from ideal reversibility. These systematic anodic shifts and increasing quasi-reversibility with higher degrees of sulfonation are fully consistent with literature reports on ruthenium polypyridyl complexes, where electron-withdrawing substituents stabilize the Ru^3+^ oxidation state and introduce kinetic barriers associated with charge transport within the MOF framework.^41,42^

As members of the UiO family of MOFs are intrinsically insulators, it is important to clarify the origin and mechanism of the currents observed during voltammetric experiments. Two limiting models are generally invoked. In one case, only the Ru sites in close proximity to the underlying electrode are electrochemically accessible, while the bulk of the film remains inactive, leading to a surface-confined response. Alternatively, the current may arise from a redox hopping mechanism, in which electrons are relayed through successive self-exchange events between neighboring redox-active centers, accompanied by ion migration for charge compensation. The process allows the redox chemistry to penetrate throughout the film, yielding behavior that resembles diffusion control. The Morris group has previously demonstrated the mechanism in related frameworks using potential-step techniques.^38,43^ To assess which pathway dominates in the synthesized films, CV was carried out over a broad range of scan rates (10–1000 mV s^−1^), enabling evaluation of the scan-rate dependence of the redox response and thus providing mechanistic insight into whether electron hopping through the framework or localized surface activity governs the observed electrochemistry.

For diffusion-controlled redox processes, the peak current typically adheres to the Randles–Sevcik equation [eqn (3) and (4)].

3 log(*i*_p_) = 0.5 log(*v*) + log[0.4463 *nFAC*(*nFD*/*RT*)^1/2^]

Conversely, if *i*_p_ arises from surface-bound redox species, it should follow eqn (3),^44^

4 *i*_p_ = *n*^2^*F*^2^*νAΓ*/4*RT*

Here, *Γ* denotes the surface coverage of electroactive species on the electrode.

**Fig. 6:**
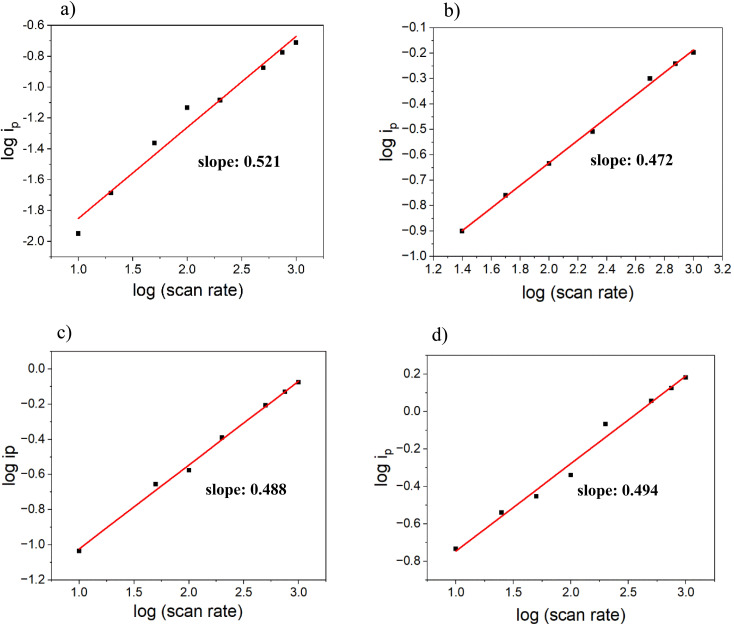
The log(*i*_p_) *versus* log(scan rate) plots for (a) RuTPY-UiO-67, (b) Ru2S, (c) Ru3S, and (d) Ru4S.

To probe whether the observed electrochemical response arises from a diffusion-driven process, the dependence of peak current on scan rate was analyzed in detail. Specifically, log–log plots of anodic peak current *versus* scan rate for the Ru^2+^/Ru^3+^ couple were constructed (Fig. 6a–d). The resulting slopes were 0.521 for RuTPY-UiO-67, 0.472 for Ru2S, 0.488 for Ru3S, and 0.494 for Ru4S, values that closely approach the theoretical slope of 0.5 predicted for diffusion-controlled charge transfer. These analyses strongly support that the Ru redox couples in these MOF films are primarily governed by the diffusion of electroactive species within the medium, rather than by processes limited to surface-bound sites.^45^ By applying eqn (2) at a scan rate of 100 mV s^−1^, *D*_app_s were determined to be (2 ± 3)×10^−10^ cm^2^ s^−1^ for RuTPY-UiO-67, (3.5 ± 0.2)×10^−9^ cm^2^ s^−1^ for Ru2S, (7 ± 2)×10^−8^ cm^2^ s^−1^ for Ru3S, and (1 ± 1)×10^−7^ cm^2^ s^−1^ for Ru4S.

#### 2.3.2 Potential step chronoamperometry

**Fig. 7:**
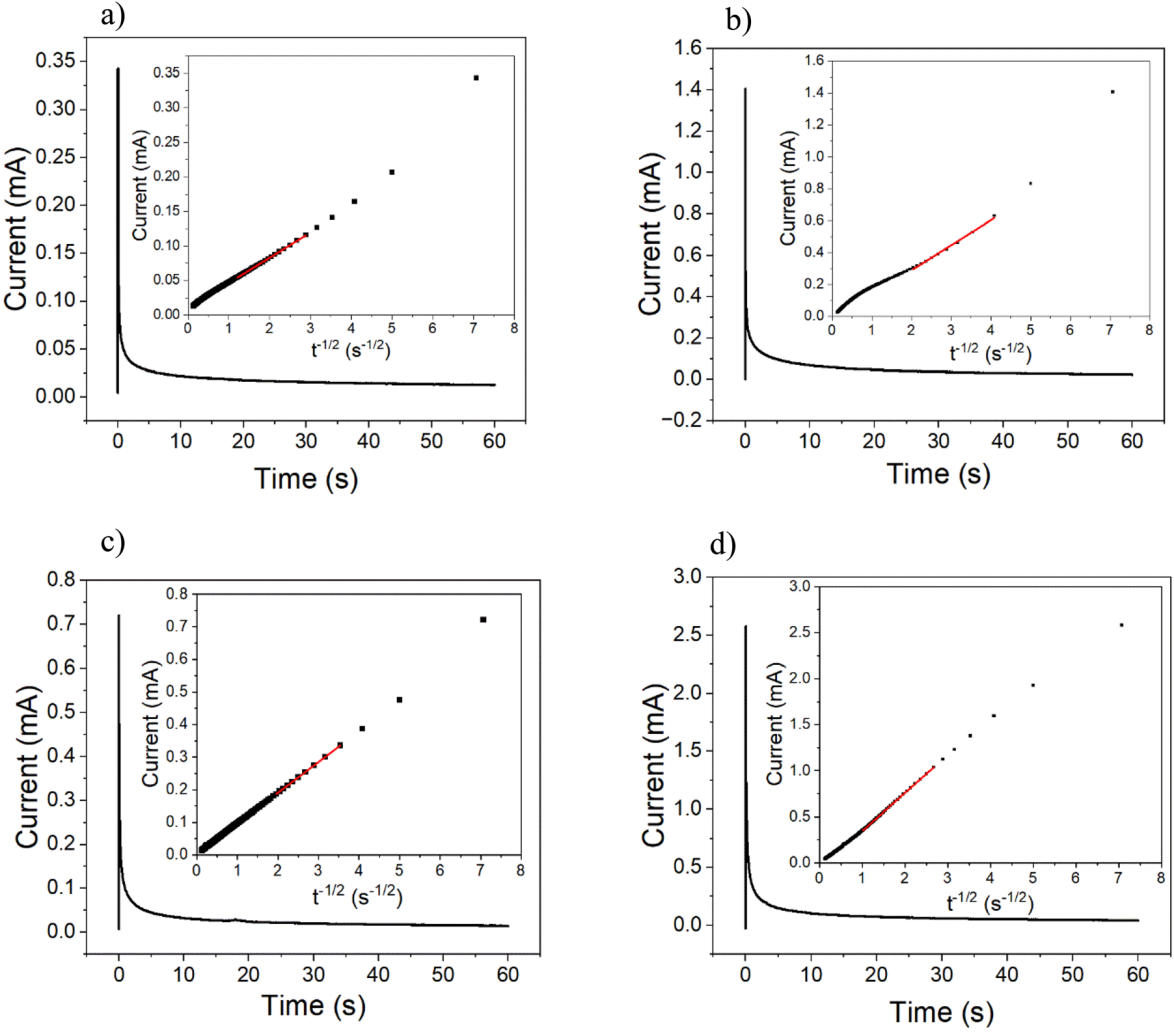
Chronoamperometric responses for (a) RuTPY-UiO-67, (b) Ru2S, (c) Ru3S, and (d) Ru4S with the insets displaying the corresponding Cottrell plots. The red traces highlight the linear regions that were used to extract the slopes for calculating *D*_app_s.

Chronoamperometric measurements were additionally employed to evaluate the *D*_app_s of the MOF films. In such experiments, the transient current response following a potential step provides direct insight into diffusional charge transport, and can be quantitatively described by the Cottrell equation (eqn (5)):5
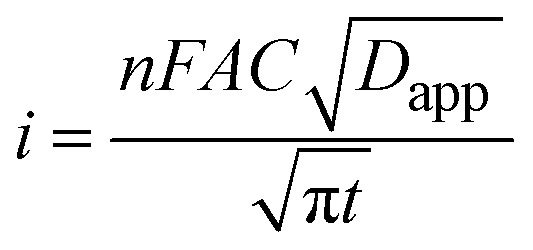
where *i* is the measured current, *n* is the number of electrons involved in the redox event, *F* is Faraday's constant, *A* is the electrode area, *C* is the concentration of the electroactive species, *D*_app_ is the apparent diffusion coefficient, and *t* is the elapsed time after the potential step. According to the relationship, a plot of current *versus* (*t*)^−1/2^ yields a straight line from which *D*_app_ can be extracted. For the present study, chronoamperometry was carried out on RuTPY-UiO-67 and its sulfonated derivatives by stepping the potential to 1800 mV *vs.* NHE. The resulting current–time profiles, shown in Fig. 7a–d, display the characteristic decay expected for diffusion-limited processes, thereby enabling reliable estimation of *D*_app_s for the MOF thin films.

The chronoamperometric responses show an initial sharp current spike due to capacitive charging and fast surface processes, followed by a decay as diffusion-controlled faradaic current dominates. According to the Cottrell equation, current decreases with *t*, so plotting current *vs. t*^−1/2^ gives a linear region after the initial transient. The linear region of the Cottrell plot was selected for *D*_app_ analysis, excluding the initial current points influenced by capacitive or non-faradaic charging and very long-time points affected by convection or vibrations. The red-highlighted linear segment demonstrates that the current response follows Cottrell behavior, confirming that semi-infinite linear diffusion governs the mass transport process. By applying the Cottrell equation, *D*_app_s were determined to be (3 ± 2)×10^−9^ cm^2^ s^−1^ for RuTPY-UiO-67, (5 ± 1)×10^−8^ cm^2^ s^−1^ for Ru2S, (1.2 ± 0.4)×10^−7^ cm^2^ s^−1^ for Ru3S, and (6 ± 3)×10^−6^ cm^2^ s^−1^ for Ru4S.

#### 2.3.3 Potential step chronocoulometry

In potential step chronocoulometry, the measured current–time response can be integrated to obtain the total charge transferred during the experiment. Plotting the charge (*q*) *versus t*^1/2^ yields a linear relationship described by the Anson equation (eqn (6)), where the slope is directly related to the apparent diffusion coefficient.6
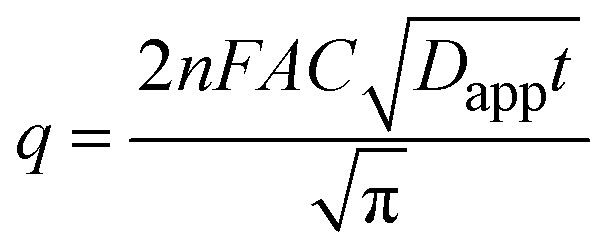


Compared to chronoamperometry, the Anson method offers higher reliability because it minimizes the effects of non-faradaic contributions such as double-layer charging and adsorption of electroactive species at the electrode surface. Additionally, since the current signal is integrated over time, the approach inherently reduces experimental noise, making it particularly useful for extracting accurate diffusion parameters in systems with low currents or noisy responses (Fig. S8). By applying the Anson equation, *D*_app_s were determined to be (1 ± 1)×10^−9^ cm^2^ s^−1^ for RuTPY-UiO-67, (3 ± 2)×10^−8^ cm^2^ s^−1^ for Ru2S, (1.1 ± 0.3)×10^−7^ cm^2^ s^−1^ for Ru3S, and (5 ± 1)×10^−6^ cm^2^ s^−1^ for Ru4S.

#### 2.3.4 Potential step chronoabsorptiometry (spectroelectrochemistry)

Potential-step chronoabsorptiometry (spectroelectrochemistry) provides an independent and robust approach to quantify apparent charge-transport rates in redox-active MOF thin films by directly tracking the time-dependent evolution of the optical response following an applied potential step. In contrast to previous methods, the technique determines *D*_app_ from the temporal change in absorbance rather than from electrochemical current, thereby avoiding uncertainties arising from capacitive charging, background currents, and parasitic side reactions. In our study, the oxidation of Ru^2+^ to Ru^3+^ within the UiO-67 films was monitored by recording the absorbance at 520 nm wavelength as a function of time immediately after application of a constant potential bias. The absorbance–time traces were normalized and plotted as a function of the square root of time (Fig. S9), and the linear region was fit using a modified Cottrell relationship (eqn (7)):7
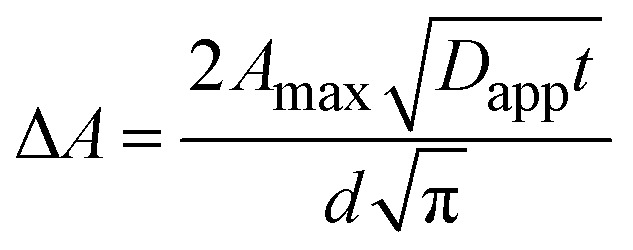
where Δ*A* is the change in absorbance, *A*_max_ is the maximum absorbance change, *t* is time (s), and *d* is the film thickness. Ru4S MOF film exhibited rapid and near complete (∼100%) optical conversion following the potential step, consistent with essentially oxidation of Ru^2+^ to Ru^3+^ throughout the film. In contrast, the parent RuTPY-UiO-67 film showed a smaller overall absorbance change, reaching a maximum conversion of only ∼57%, indicating that a fraction of Ru centers in the native framework is electrochemically inaccessible. The analysis was performed across 12 various independently prepared films, yielding *D*_app_ of (3 ± 1)×10^−9^ cm^2^ s^−1^ for RuTPY–UiO-67, (1.6 ± 0.4)×10^−8^ cm^2^ s^−1^ for Ru2S, (2 ± 2)×10^−7^ cm^2^ s^−1^ for Ru3S, and (7.8 ± 0.8)×10^−6^ cm^2^ s^−1^ for Ru4S. These values follow the same monotonic trend observed from current-based analyses (CV, CA, and CC) and fall within the combined experimental uncertainty of those methods, supporting a consistent description of charge transport across techniques. A key advantage of the spectroelectrochemical approach is that, unlike the potential step Cottrell and Anson analyses (eqn (5) and (6)), eqn (7) does not require explicit knowledge of the concentration of electroactive Ru centers, as the normalization by *A*_max_ intrinsically accounts for variations in redox-site density. As a result, the spectroelectrochemical method provides a particularly reliable measure of *D*_app_ for heterogeneous MOF films where incomplete electrochemical accessibility or uncertainties in loading can complicate current-derived analyses. The normalization, however, renders the extracted diffusion coefficient sensitive to the accurately measured film thickness, as illustrated in Fig. S2.

## 3. Analysis of the obtained *D*_app_s and the role of sulfonic acid groups

Table 1 summarizes the *D*_app_s determined by different methods for RuTPY-UiO-67 and its sulfonated analogs. The values obtained by the various techniques are consistent within experimental error, suggesting that the true diffusion coefficients are well represented by the averaged estimates of (2 ± 2) × 10^−9^ cm^2^ s^−1^ for RuTPY-UiO-67, (3 ± 2) × 10^−8^ cm^2^ s^−1^ for Ru2S, (1.3 ± 0.5) × 10^−7^ cm^2^ s^−1^ for Ru3S, and (5 ± 3) × 10^−6^ cm^2^ s^−1^ for Ru4S.

**Table 1:** Compilation of *D*_app_ values (cm^2^ s^−1^) for MOFs as determined by various methods

MOF thin film	CV	Cottrell (electrochemical)	Cottrell (spectroelectrochemical)	Anson	Average
RuTPY-UiO-67	(2 ± 3) × 10^−10^	(3 ± 2) × 10^−9^	(3 ± 1) × 10^−9^	(1 ± 1) × 10^−9^	(2 ± 2) × 10^−9^
Ru2S	(3.5 ± 0.2) × 10^−9^	(5 ± 1) × 10^−8^	(1.6 ± 0.4) × 10^−8^	(3 ± 2) × 10^−8^	(3 ± 2) × 10^−8^
Ru3S	(7 ± 2) × 10^−8^	(1.2 ± 0.4) × 10^−7^	(2 ± 2) × 10^−7^	(1.1 ± 0.3) ×10^−7^	(1.3 ± 0.5) × 10^−7^
Ru4S	(1 ± 1) × 10^−7^	(6 ± 3) × 10^−6^	(7.8 ± 0.8) × 10^−6^	(5 ± 1) × 10^−6^	(5 ± 3) × 10^−6^

Several studies have shown that adding sulfonic acid groups to porous materials can greatly improve ion transport. For instance, Xu *et al.* found that UiO-66-SO_3_H membranes allowed cations to move more easily through the pores because the deprotonated sulfonate sites interacted strongly with hydrated metal ions.^46^ Lee *et al.* later reported a similar effect in sulfonated covalent organic frameworks, where the fixed anionic sites formed continuous channels that helped Na^+^ and K^+^ ions hop through the structure.^47^ The enhanced charge-transfer behavior observed for our sulfonated frameworks is attributed to ion–framework interactions rather than any redox activity of the substituent (no SO_3_H redox features were observed as shown in Fig. S10). The presence of –SO_3_H creates anionic sites that attract Li^+^ ions from the LiClO_4_ supporting electrolyte, reducing tight Li^+^–ClO_4_^−^ ion pairing and changing the local electrostatic environment inside the pores. As a result, anions move more freely, and electron hopping between redox sites becomes more efficient, leading to the higher apparent diffusion coefficients seen for the sulfonated materials.

**Fig. 8:**
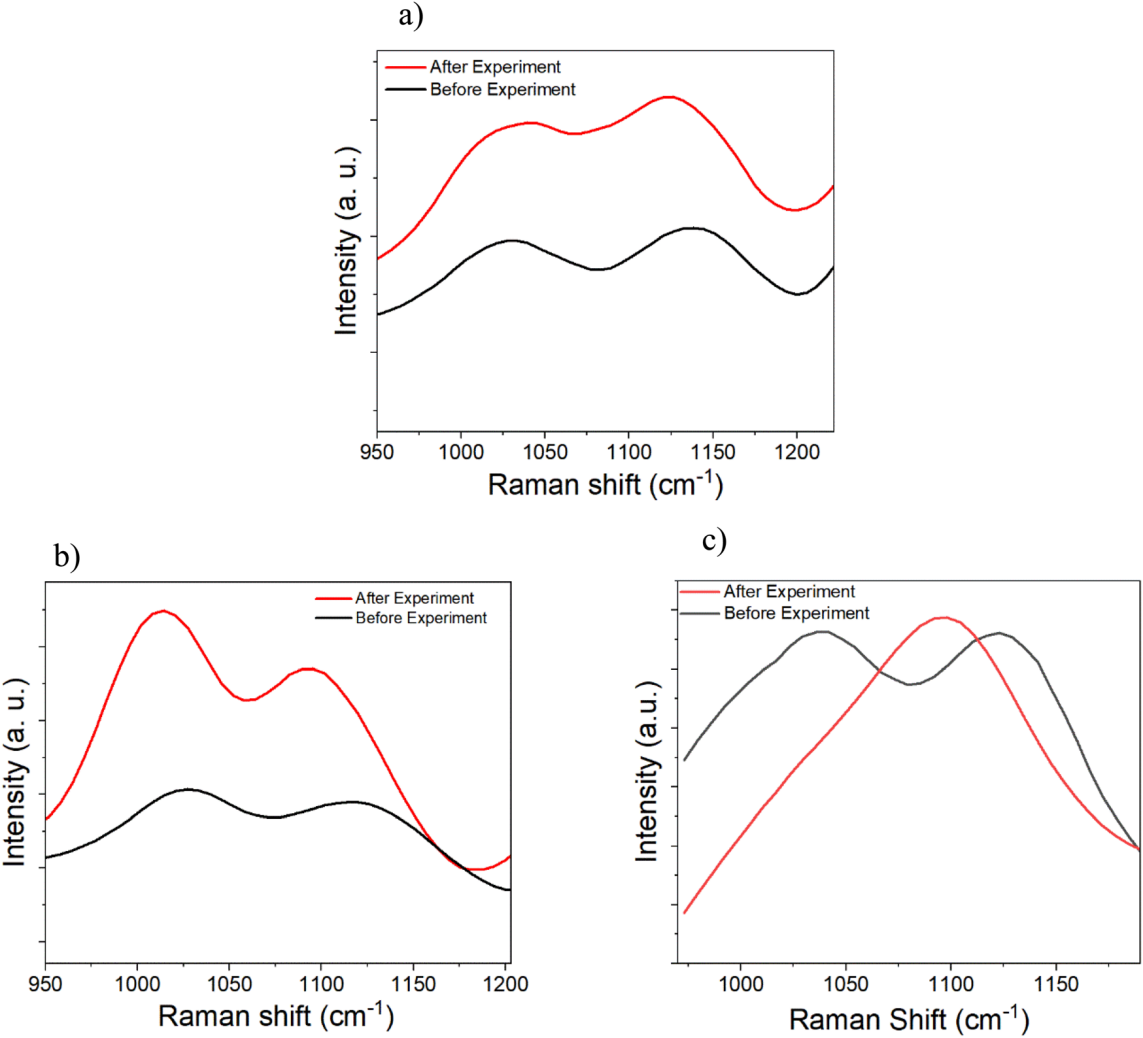
Raman Spectra of (a) Ru2S, (b) Ru3S, and (c) Ru4S MOFs before (black) and after (red) electrochemistry experiments.

To provide additional evidence for the proposed mechanism of interaction between Li^+^ ions and the sulfonated MOF framework, Raman spectroscopy was utilized to examine possible structural and coordination modifications occurring for sulfonate functional group before and after electrochemical testing in an electrolyte solution containing LiClO_4_ (Fig. 8). Raman spectroscopy was chosen because of its high sensitivity to vibrational modes of specific functional groups, particularly sulfonate groups, which are key sites for ion interaction. The technique enables the detection of subtle changes in the local chemical environment, such as shifts in bond strength, that may arise from the direct Li^+^ binding. Within the sulfonated MOF, the Raman spectrum distinctly displayed the stretching vibrations of the sulfonate groups, appearing at characteristic wavenumbers around ∼1025 cm^−1^ and ∼1140 cm^−1^.^48^ The observed Raman red shifts (∼12 cm^−1^ for Ru2S, ∼32 cm^−1^ for Ru3S, and ∼38 cm^−1^ for Ru4S) indicate a clear trend of increasing Li^+^–SO_3_^−^ interaction strength and concomitant ion-pair disruption in the order: Ru4S > Ru3S > Ru2S. Mechanistically, Li^+^ is a small, hard, highly polarizing cation that coordinates to sulfonate oxygens and withdraws electron density from the S–O bonds, which lowers bond order and produces a red shift of the S–O stretching modes. The behavior is well documented for Li-sulfonates and related ion pairs.^49–51^ The much larger shifts for the Ru3S and Ru4S MOFs are consistent with greater site accessibility and the possibility of multi-site or cooperative coordination in Ru4S that increases cumulative S–O weakening, whereas Ru2S introduces steric/electronic constraints that limit Li^+^ access or enforce asymmetric, localized binding and thus a smaller spectral perturbation. Experimentally, sulfonated polymers and small-molecule sulfonates show exactly the same pattern where ion exchange with Li^+^ produces pronounced red shifts and broadening of the asymmetric S–O band while the symmetric feature becomes weaker or merges into a broadened envelope, which together are taken as signatures of stronger cation coordination and partial/complete ion-pair breaking. These spectral assignments and the correlation of red shift magnitudes with the degree of Li^+^ binding are therefore consistent with prior Raman studies and provide a quantitative basis to argue that Ru4S undergoes the most extensive Li^+^ coordination and ion-pair breaking, the Ru3S an intermediate amount, and the sterically hindered Ru2S the least. Such analysis not only supports the Li^+^ interaction hypothesis but also provides a deeper understanding of the framework's structural resilience and functional behavior under operational conditions.

## 4. Conclusions

In this work, we demonstrated how sulfonic acid functionalization profoundly influences charge transport and electrochromic performance in UiO-67-based MOFs. By systematically varying the number and position of –SO_3_H groups on biphenyl dicarboxylate linkers, we established clear structure–property relationships linking substitution pattern to ion mobility and redox efficiency. Spectroelectrochemical analysis revealed that RuTPY-UiO-67 suffered from incomplete redox conversion (∼57%) and limited charge transport. All sulfonated frameworks outperformed the parent RuTPY-UiO-67, with improvements attributed to sulfonate-mediated ion pair dissociation and enhanced counterion transport. Notably, the tetra-substituted Ru4S MOF displayed the highest *D*_app_ (5 ± 3) × 10^−6^ cm^2^ s^−1^, along with complete (∼100%) redox conversion, high coloration efficiency (641 cm^2^ C^−1^), a large optical contrast (89.2%), and achieved the fastest and most reversible electrochromic response, with a coloration time of 1.09 s and a bleaching time of 1.5 s. To the best of our knowledge, this represents the highest *D*_app_ reported for a redox-active MOF to date. Complementary Raman spectroscopy confirmed strong cation–sulfonate interactions, supporting a mechanism where charged functional groups promote efficient redox hopping throughout the framework. Collectively, these results transform sulfonation from a qualitative design motif into a quantitative, structure–function parameter for engineering charge transport in electroactive MOFs. More broadly, the work provides mechanistic insight into how chemical patterning of porous frameworks can be exploited to control ion–electron transport, offering a general strategy for the rational design of high-performance MOF-based electrochromic, energy-storage, and optoelectronic devices.

## Author contributions

SB performed ligand synthesis and characterization, carried out electrochemical, spectroelectrochemical, and Raman experiments, analyzed the data, and drafted the manuscript. LP contributed to MOF synthesis. ZD acquired the SEM images. AJM conceived and designed the research, reviewed the manuscript, and obtained funding.

## Conflicts of interest

A patent application covering aspects of the materials and methods described in this work has been submitted.

## Supplementary Material

SC-017-D6SC02965E-s001

## Data Availability

Crystallographic data for the reference UiO-67 framework was obtained from the Cambridge Structural Database (CCDC 1021002) *via* ref. 52. The data supporting this article have been included as part of the supplementary information (SI). Supplementary information: materials; characterization methods; synthesis of the chromophore, sulfonated linkers; cross-section SEM images; electronic absorption spectra of Ru2S, Ru3S, and Ru4S; Anson plots and cyclic voltammograms of MTV MOF thin films (including Ru2S, Ru3S, and Ru4S without chromophore); and literature comparisons of MOF-based electrochromic performance and diffusion coefficients. See DOI: https://doi.org/10.1039/d6sc02965e.
