# Supplementary material for: Tunable redox hopping charge transport and electrochromism in multivariate MOFs: effects of substitution patterns and number of sulfonic acid groups
Source: Chem Sci. 2026 Jun 4;17(32):15490–502. doi: 10.1039/d6sc02965e (PMC13322743; doi:10.1039/d6sc02965e)
Supplement: Supplementary file 1 [file SC-017-D6SC02965E-s001.pdf]

Supplementary Information (SI) for Chemical Science.
This journal is © The Royal Society of Chemistry 2026

Supporting Information

for

Tunable redox hopping charge transport and electrochromism in multivariate MOFs: effects of substitution patterns and number of sulfonic acid groups

Sumanta Basak [a], Laura Prince [a], Zhengyu Du [b], and Amanda J. Morris [a,b] *

[a]Department of Chemistry, Virginia Polytechnic Institute and State University, Blacksburg, Virginia 24061, United States

[b]Macromolecules Innovation Institute, Virginia Polytechnic Institute and State University, Blacksburg, Virginia 24061, United States

Contents	Page
Materials	**S2**
Characterization methods	**S2-S4**
Synthesis of chromophore, sulfonated linkers, and MTV MOFs	**S4-S7**
Cross-section SEM images of MTV MOF thin films	**S8**
Electronic absorption and transmittance spectra of Ru2S, Ru3S, Ru4S	**S9-10**
Anson plots of the MTV MOF thin films	**S12**
CVs of Ru2S, Ru3S, Ru4S without chromophore	**S13**
Overview of reported framework-based electrochromic materials in the literature compared with the present study.	**S14-15**
Overview of reported diffusion coefficients and cycling stability of MOF in the literature compared with the present study.	**S15-17**

*Email: ajmorris@vt.edu

1. Materials

The following chemicals were all purchased from AmBeed (Buffalo Grove, IL, USA): $RuCl_3$*$3H_2O$ (97%), 2,2′;6′,2″-terpyridine (97%), 2,2′-bipyridine-5,5′-dicarboxylic acid (97%), 4-aminotoluene-3-sulfonic acid (98%), 4,4′-dimethyl-1,1′-biphenyl (97%). Ethanol was obtained from Decon Labs Inc. (Swedeland, PA, USA). LiCl, potassium permanganate, 4,4′-biphenyl dicarboxylic acid, and glacial acetic acid were purchased from Oakwood Chemical (Estill, SC, USA). Triethylamine (99.5%), copper powder (99.99% trace metals basis), lithium perchlorate (99.99% trace metals basis), potassium hydroxide (ACS reagent, ≥85%, pellets), fuming sulfuric acid, Deuterium oxide with 99.9 atom% D and 0.05 wt. % 3-(trimethylsilyl)propionic-2,2,3,3-d_4 acid, sodium salt, and $ZrCl_4$ (≥99.9% trace metals basis) were obtained from Sigma-Aldrich (St. Louis, MO, USA). The following chemicals were purchased from Thermo Fisher Scientific (Waltham, MA, USA): acetonitrile, potassium nitrite, potassium iodide (99%), sulfuric acid, N, N-dimethylformamide (DMF, ≥99.8%), acetone, sodium hydroxide, and hydrochloric acid. FTO slides were purchased from Hartford Glass Co., Inc. (Hartford City, IN, USA).

2. Characterization methods

2.1 Nuclear magnetic resonance (NMR) spectroscopy

All ^{1}H experiments were performed on the JEOL ECZ 400 (400 MHz). ^{13}C experiments were performed on the Bruker Avance Neo 600 (600 MHz). For quantitative analysis, 64 scans with a 10 s relaxation delay were used.

2.2 Powder X-ray diffraction (PXRD)

PXRD patterns for the MOF films were collected on a Bruker D8 Advance Wide Angle X-Ray diffractometer with a Cu k_α (λ = 0.1541 nm) radiation source generated at 40 kV and 40 mA. The measurements were collected from 2θ values of 2° to 60° with a resolution of 0.02° at a rate of 0.25° per minute.

2.3 Absorbance measurements

Absorbance measurements were taken on a Cary UV-Vis-NIR spectrophotometer from Agilent Technologies. Spectral scan measurements were taken in MeCN from 800 nm to 200 nm at a scan rate of 600 nm per minute.

For the kinetic studies, absorbance at a chosen wavelength was monitored with data points recorded every 0.033 s. The MOF film was mounted in a glass cuvette, aligned so that the beamline passed directly through it. The cuvette was filled with 0.1 M $LiClO_4$ in MeCN, and an Ag/$AgNO_3$ reference electrode, together with a platinum counter electrode, was inserted into the cell without obstructing the beam path. A standard spectral scan was used to identify the λ_{max}, while a cyclic voltammogram recorded at 100 mV/s provided the redox potential. Kinetics were then probed using a cyclic step chronoamperometry experiment, in which 1800 mV vs. NHE was applied, followed by the open circuit potential (OCP).

2.4 Scanning electron microscopy (SEM)

SEM images were collected with a LEO 1550 field-emission scanning electron microscope (Carl Zeiss, Oberkochen, Germany) at 5.0 kV and a 7.0 mm working distance.

2.5 Raman spectroscopy

Raman spectra of the MOF thin films were obtained through the excitation of a 532 nm laser. Raman signals were collected with a CCD camera (Horiba XploRA PLUS). The measurements were conducted under ambient conditions. The Raman spectra were obtained using a 10x objective lens.

2.6 Electrochemical measurements

Electrochemical characterization was carried out using a Pine Instruments WaveNow potentiostat in a three-electrode configuration, with the MOF-coated FTO slide serving as the working electrode, an $Ag/AgNO_3$ reference electrode, and a platinum mesh counter electrode. All measurements were performed in 0.1 M $LiClO_4$/MeCN electrolyte solution. Cyclic voltammetry was conducted by sweeping the potential to 2000 mV vs. NHE, extending beyond the $Ru^{2+/3+}$ redox couple. At least six consecutive scans were recorded to ensure stabilization of the current response, and the final cycle was used for analysis. The scan rate was systematically varied between 10 and 1000 mV/s to provide the data required for diffusion coefficient determination.

3. Synthesis of the chromophore and sulfonated linkers

3.1 [Ru(tpy)(dcbpy)Cl]PF_6 synthesis

[Ru(tpy)(dcbpy)Cl]PF_6 (where tpy = 2,2′ ;6′ ,2″-terpyridine and dcbpy = 2,2′ -bipyridine-5,5′-dicarboxylic acid) was synthesized according to a previously developed synthetic method.[1] $RuCl_3$ (156.8 mg, 0.7 mol) was mixed with 2,2′;6′,2″- terpyridine (172.8 mg, 0.7 mmol) in 50 mL of ethanol. The mixture was refluxed for 4 hours at 100 °C, and a brown solid was filtered (Ru(tpy)Cl_3). Ru(tpy)Cl_3 (1 g, 2.27 mmol) was then added to 2,2′ -bipyridine-5,5′ -dicarboxylic acid (509 mg, 2.08 mmol), LiCl (100 mg, 2.35 mmol) and triethylamine (1 mL) in 3: 1 ethanol: water (100 mL). The reaction was refluxed for 16 hours at 100 °C. The reaction mixture was

filtered in hot condition and reduced to 25 mL by rotary evaporation and 15 mL of saturated NH_4PF_6 solution was added. 1 M HCl was added dropwise until [Ru(tpy)(dcbpy)Cl]PF_6 was precipitated. ^{1}H NMR: (400 MHz, DMSO-d_6): δ 7.31 (2 H, m), 7.61 (1 H, s), 7.69 (2 H, m), 7.99 (2 H, t), 8.12 (d,1 H, d), 8.28 (1 H, t), 8.69 (3 H, m), 8.79 (1 H, d), 8.85 (2 H, d), 9.15 (1 H, d), 10.6 (1 H, d).

3.2 Synthesis of sulfonated linkers

3.2.1 Synthesis of Biphenyl-2,2′-disulfonyl-4,4′-dicarboxylic acid

For the synthesis of Biphenyl-2,2′-disulfonyl-4,4′-dicarboxylic acid, a previously developed method was used.[2] Potassium nitrite was slowly added to a solution containing 4-aminotoluene-3-sulfonic acid at 3-5 °C and was stirred for 2 hours. The solid wet mass produced was added to a mixture of potassium iodide and 25% sulfuric acid and was heated. The resulting precipitate (2-iodo-5-methylbenzene sulfonic acid) was dissolved in a solution containing copper sulfate and heated for 15 mins at 90 °C. Copper powder was then added, and the solutions were allowed to stir for 5 hours. After filtration, the product was recrystallized. The product (4,4'-dimethyl-biphenyl-2,2'-disulfonic acid (11.0 mmol) was then placed in a solution containing potassium hydroxide. The solution was then heated to 50 °C, and potassium permanganate was added slowly over a period of 2 hours, followed by stirring for an additional 20 hours. After filtration, the product 2,2'-disulfonyl-4,4'-biphenyldicarboxylic acid was collected. ^{1}H NMR: (400 MHz, D_2O): δ 8.52 (2 H, s), 8.25 (2 H, d), 7.75 (2 H, dd).

3.2.2 Synthesis of Biphenyl-3,3′-disulfonyl-4,4′-dicarboxylic acid

Biphenyl-3,3′-disulfonyl-4,4′-dicarboxylic acid was synthesized following a literature procedure.[3] 4,4′-Dimethylbiphenyl (10 g) was stirred in concentrated sulfuric acid (30 mL; 98%) at 90 °C for 8 h. After cooling, acetonitrile and dichloromethane were added to precipitate 3,3′-disulfonyl-4,4′-

dimethylbiphenyl, which was dried and dissolved in 75mL aqueous NaOH (pH 12). $KMnO_4$ (12 g) was added, and the mixture was heated at 90 °C overnight. The resulting brown solid was filtered off, and the filtrate was acidified with 12 M HCl and refrigerated for 10 h to precipitate the product, which was collected and washed with 1 M HCl. ^{1}H NMR: (400 MHz, DMSO-d_6): δ 8.10 (2 H, s), 7.85 (4H, s).

3.2.3 Synthesis of Biphenyl-3,3',5,5'-tetrasulfonyl-4,4'-dicarboxylic acid

The compound was synthesized following a previously reported procedure.[4] For the synthesis of Biphenyl-3,3',5,5'-tetrasulfonyl-4,4'-dicarboxylic acid, 4,4′-dimethylbiphenyl (500 mg) was stirred with 10 mL of fuming H_2SO_4 at 90 °C. The reaction mixture was subsequently added to acetonitrile, yielding a white precipitate, which was washed sequentially with acetonitrile and dichloromethane to afford Biphenyl-3,3',5,5'-tetrasulfonyl-4,4'-dimethyl. The intermediate product was dissolved in 50 mL of aqueous NaOH and heated at 50 °C. Potassium permanganate was added slowly over 2 hours, and the mixture was stirred for an additional 20 hours. After filtration, the final product, Biphenyl-3,3',5,5'-tetrasulfonyl-4,4'-dicarboxylic acid, was obtained by acidification with 12 M HCl. ^{1}H NMR: (400 MHz, D_2O): δ 8.38 (4 H, s). ^{13}C NMR (151 MHz, D_2O): δ 165.50, 140.67, 138.55, 130.44, 128.73. ESI-MS(+) (H_2O): Calculated for 561.384, found: 561.399.

Safety considerations: Fuming sulfuric acid is an extremely hazardous chemical that requires strict safety precautions due to its highly corrosive and strongly oxidizing nature. It should always be handled in a well-ventilated fume hood while wearing appropriate personal protective equipment, including acid-resistant gloves, a lab coat or apron, eye protection, and, if necessary, a respirator.

4. MTV MOF Synthesis

For the synthesis of RuTPY-UiO-67, a previously developed synthetic procedure was used.[5] $ZrCl_4$ (58.25 mg, 0.25 mmol), biphenyl dicarboxylic acid (50.8 mg, 0.21 mmol), and [Ru(tpy)(dcbpy)Cl]PF_6 (32.6 mg, 0.043 mmol) were added to a 6-dram vial along with DMF (10 mL) and glacial acetic acid. The solution was sonicated, and a clean FTO slide was placed inside with the conductive side facing down. The MOF was then grown solvothermally in an oven at 120 °C for 1 day. After heating, the films were removed, rinsed with DMF, and stored in fresh water for 24 hrs. Ru2S, Ru3S, and Ru4S MTV MOF thin films were prepared using the same procedure as above; however, the BPDC ligand was substituted for corresponding sulfonated ligands.

a)

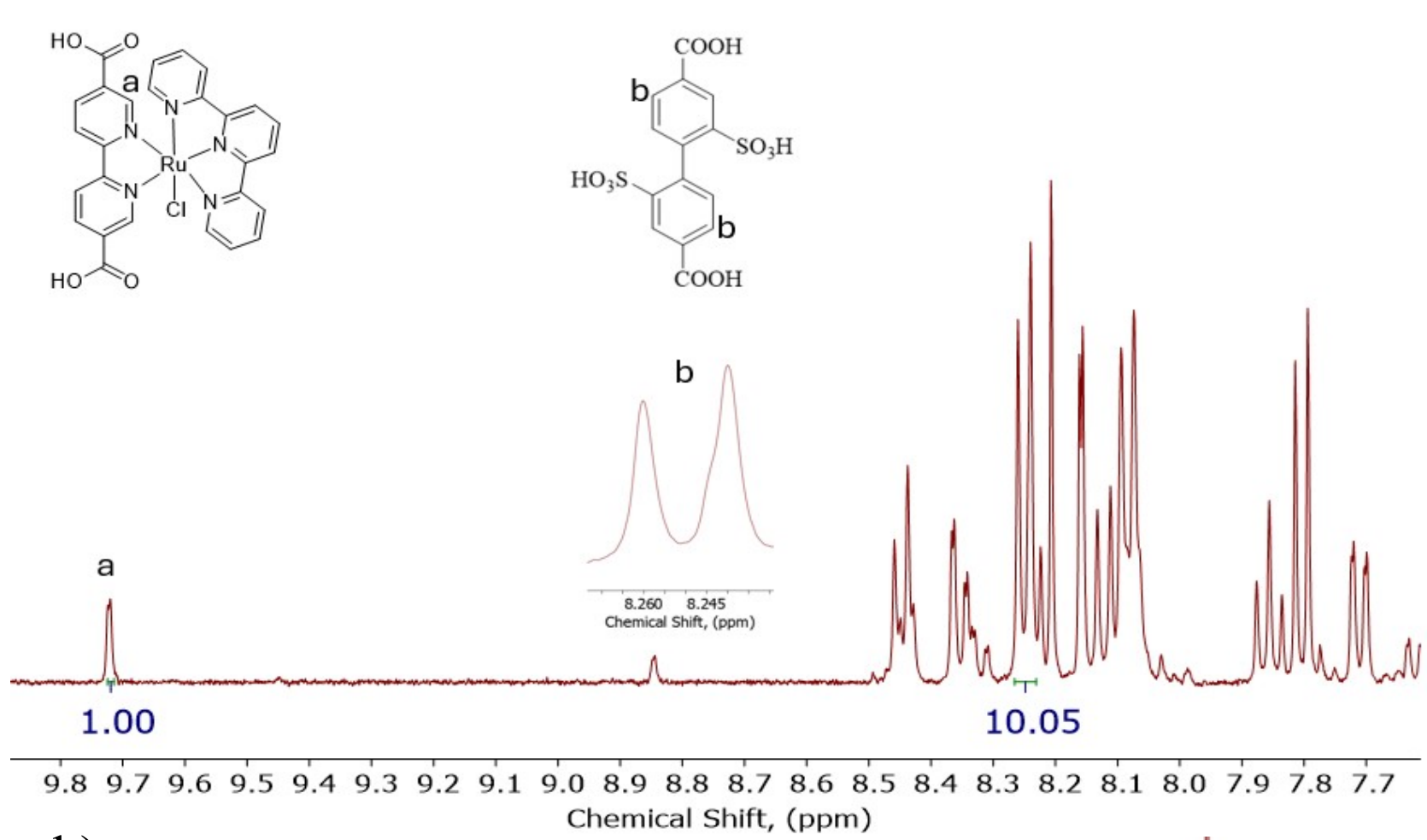

b)

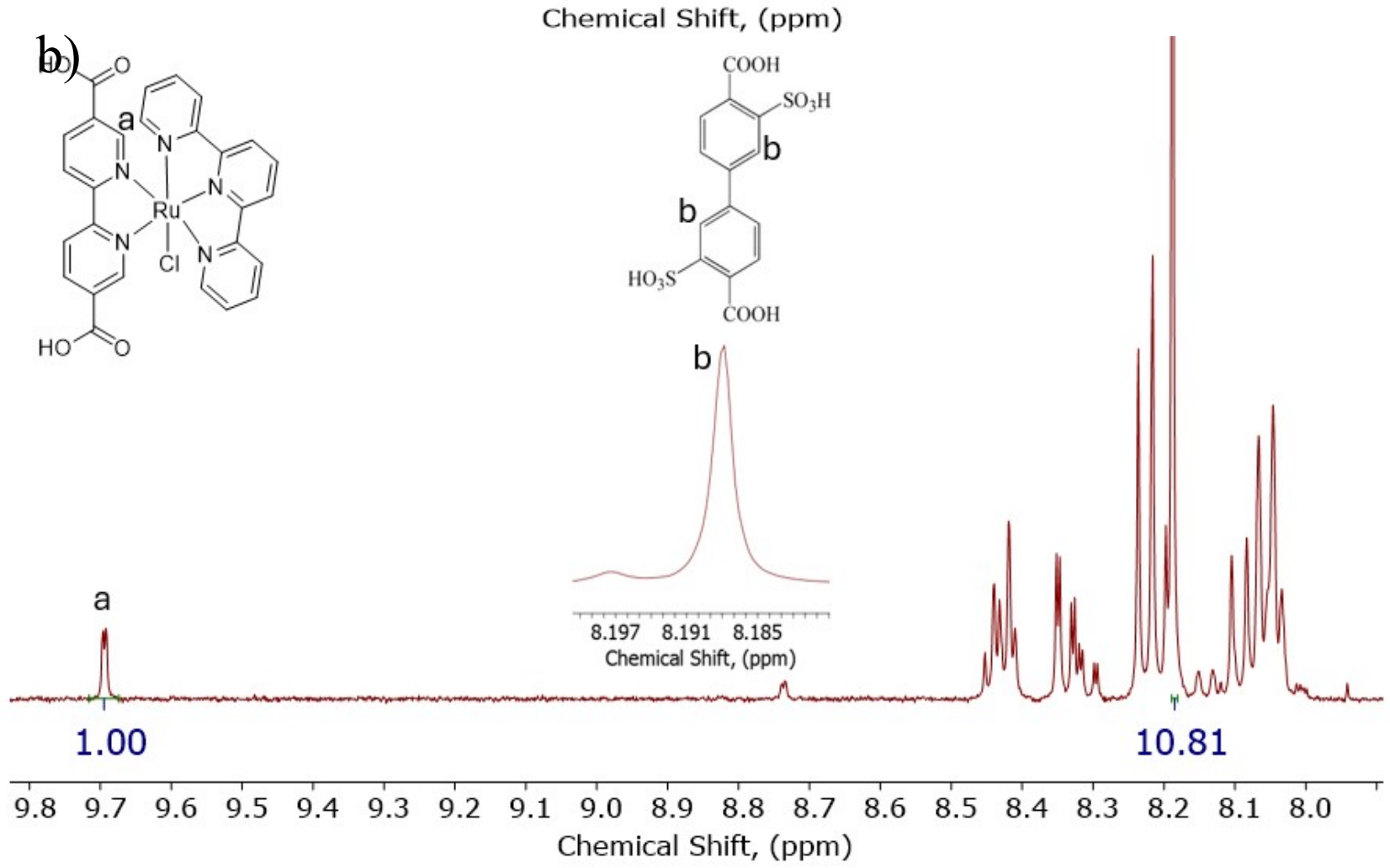

c)

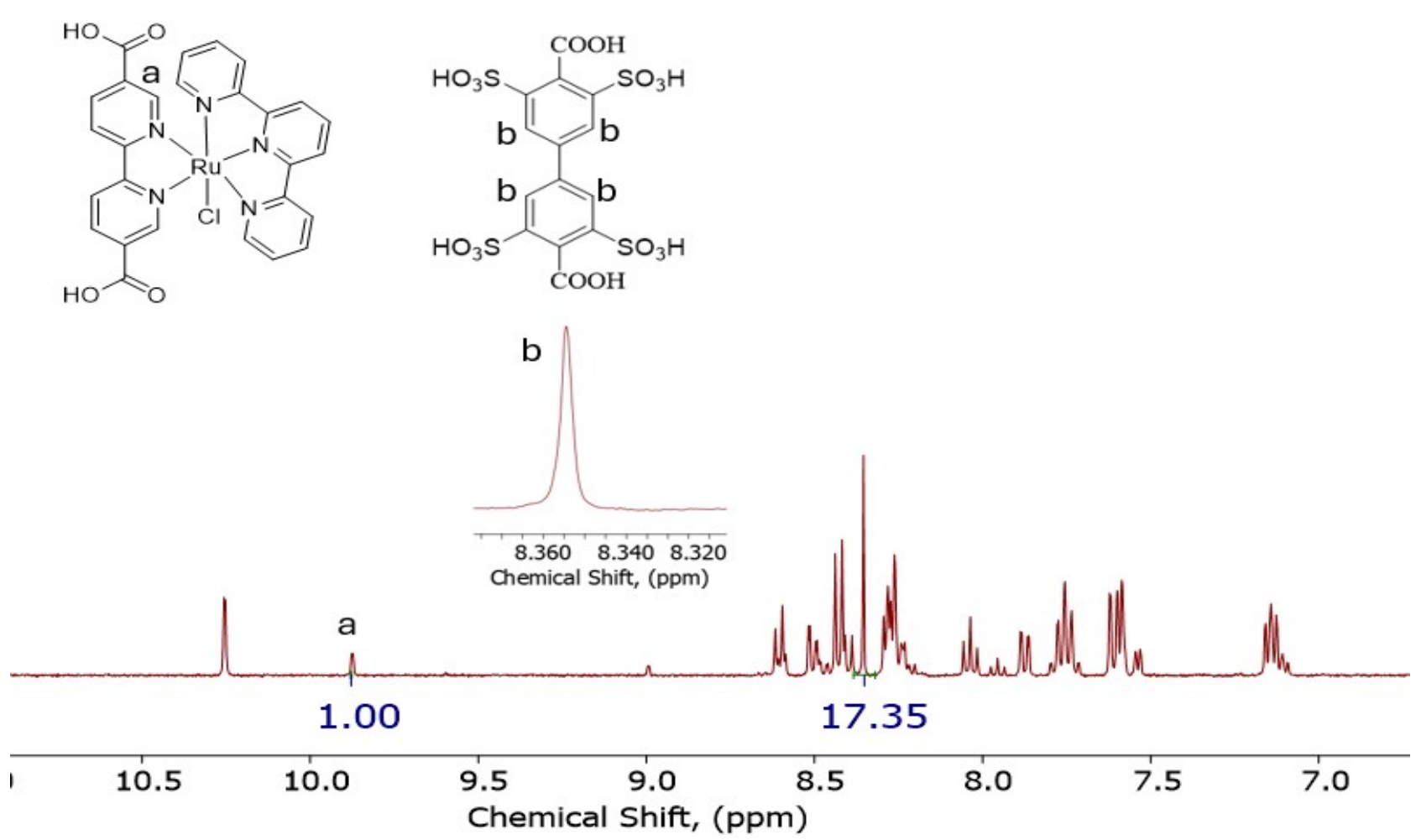

Fig. S1 ^{1}H NMR spectrum of digested (a) Ru2S, (b) Ru3S, and (c) Ru4S MOFs.

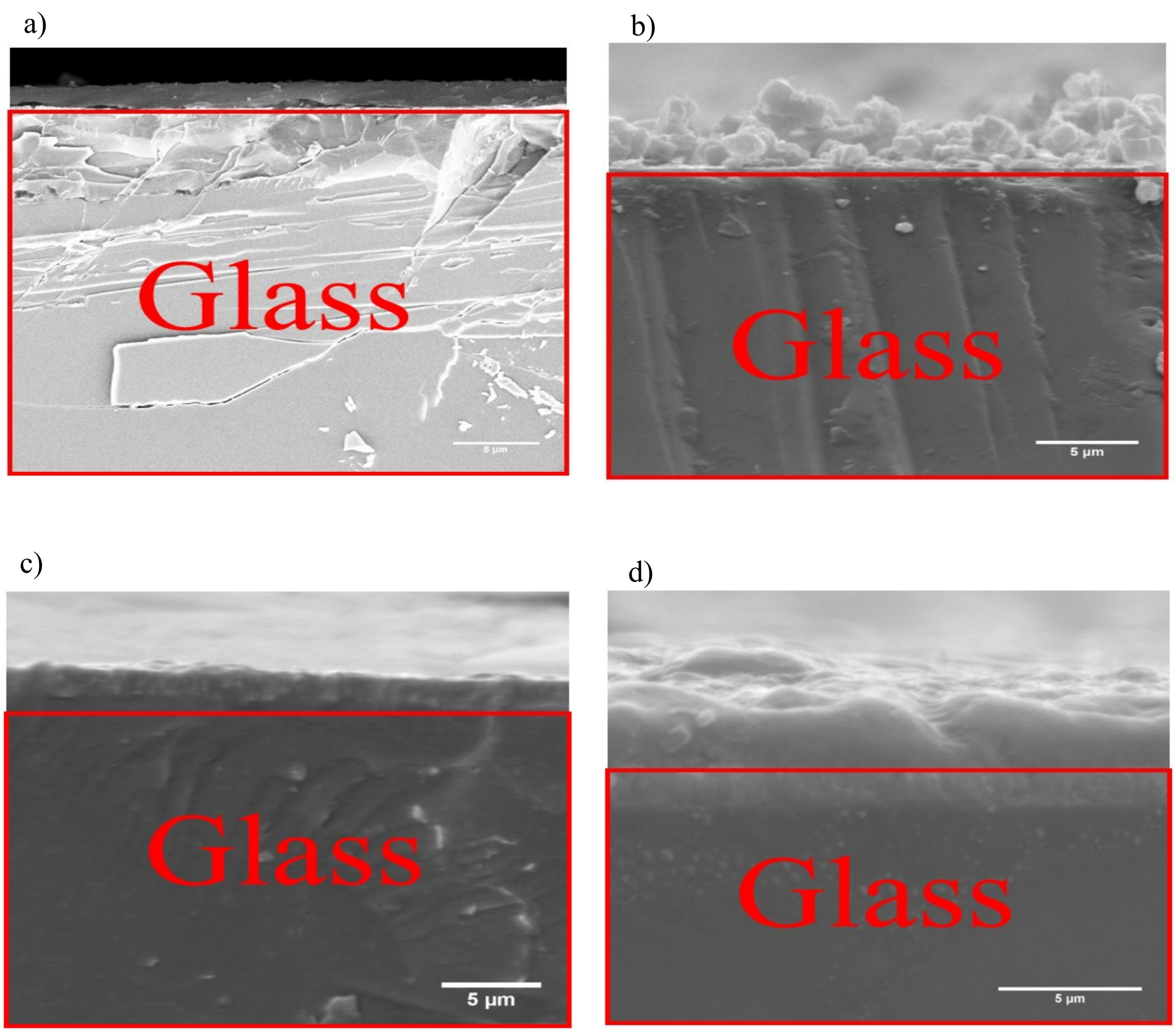

Fig. S2 Cross-section SEM images of (a) RuTPY-UiO-67, (b) Ru2S, (c) Ru3S, and (d) Ru4S MOF thin films on FTO. The "Glass" label denotes the bulk substrate below the thin fluorine-doped tin oxide (FTO) layer. The FTO layer serves as the conductive electrode for the MOF film. Average film thicknesses were determined using ImageJ software.

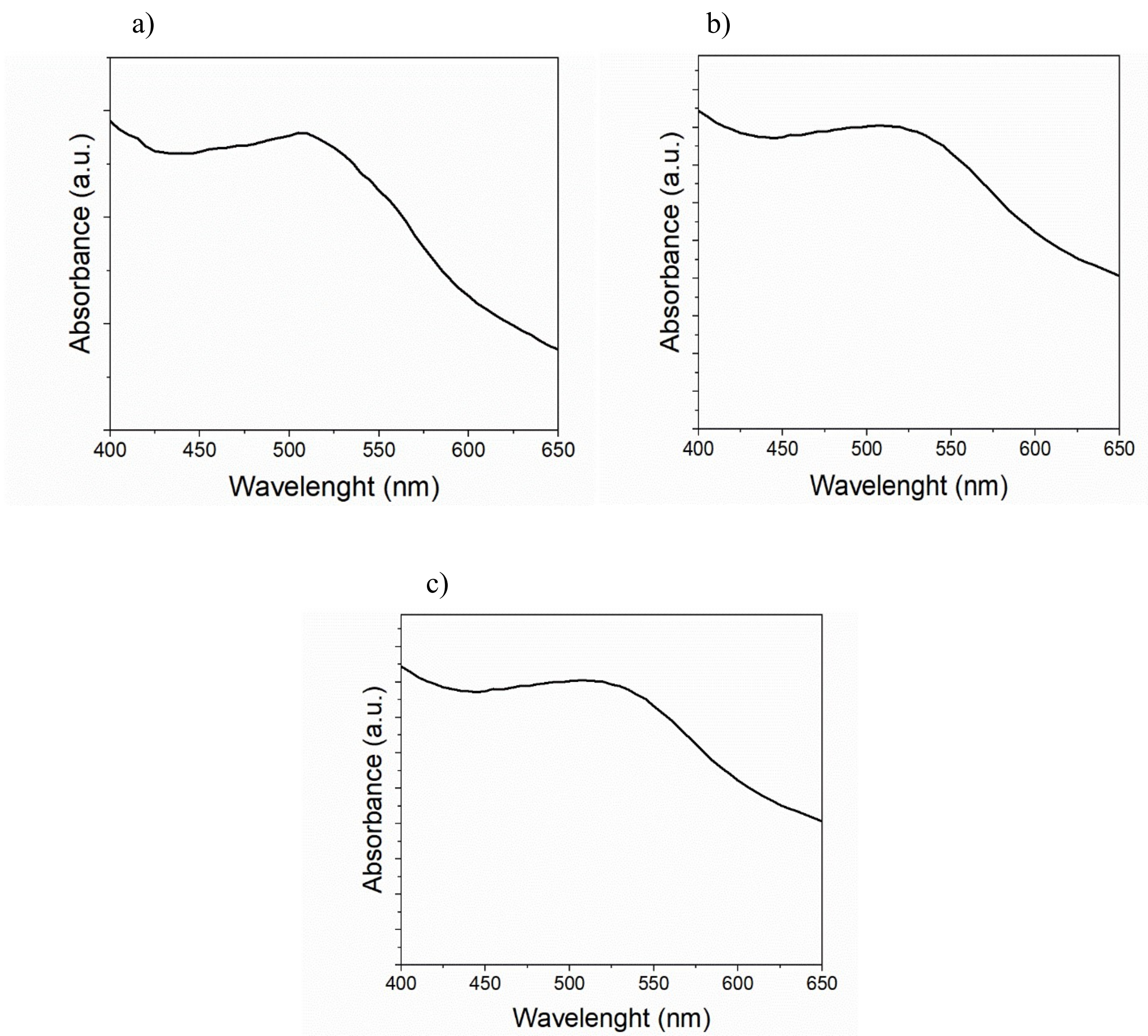

Fig. S3 The electronic absorption spectra of (a) Ru2S, (b) Ru3S, and (c) Ru4S.

a) b)

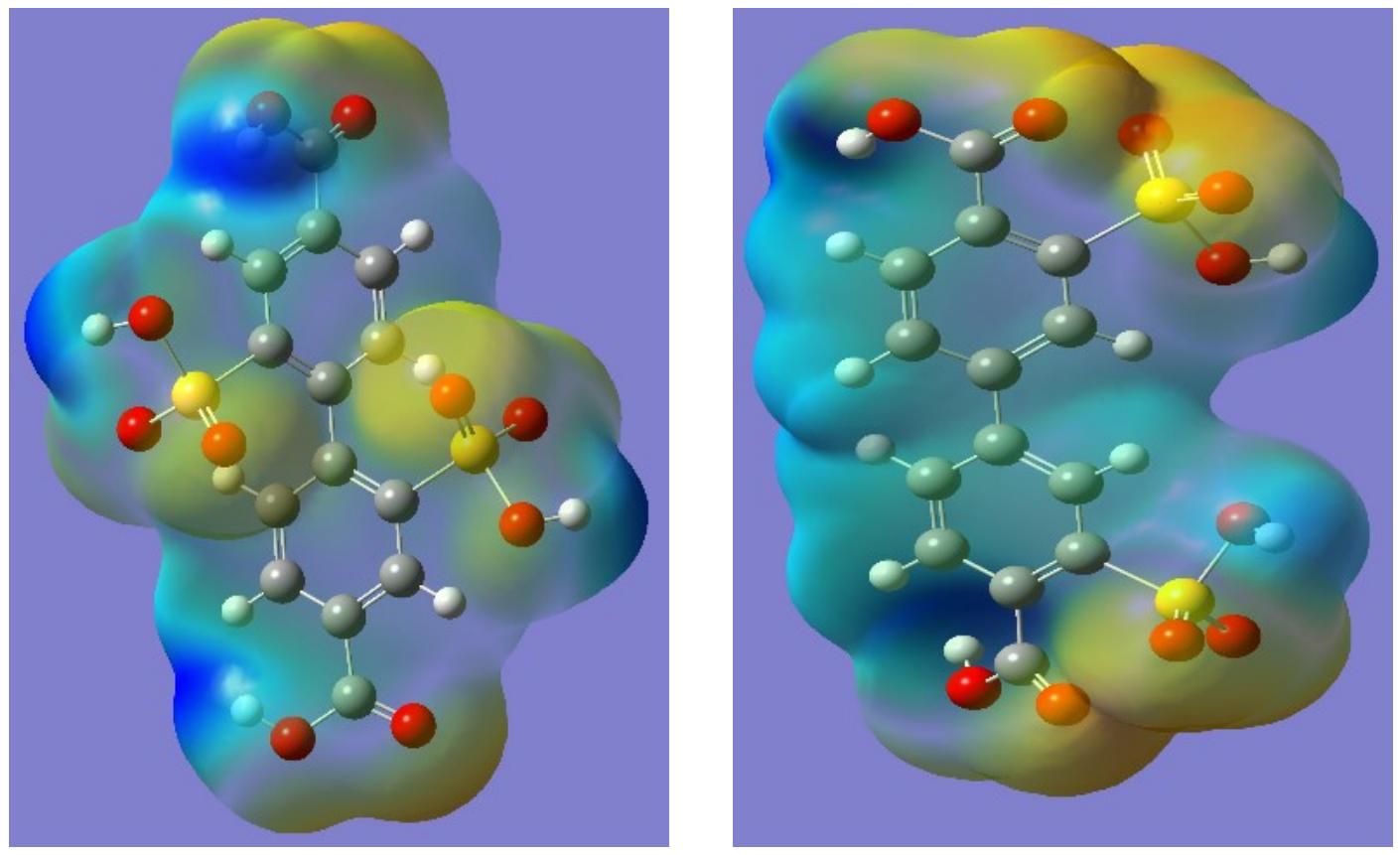

Fig. S4 Electrostatic potential maps of (a) 2,2′- and (b) 3,3′-disulfonated biphenyl linkers.

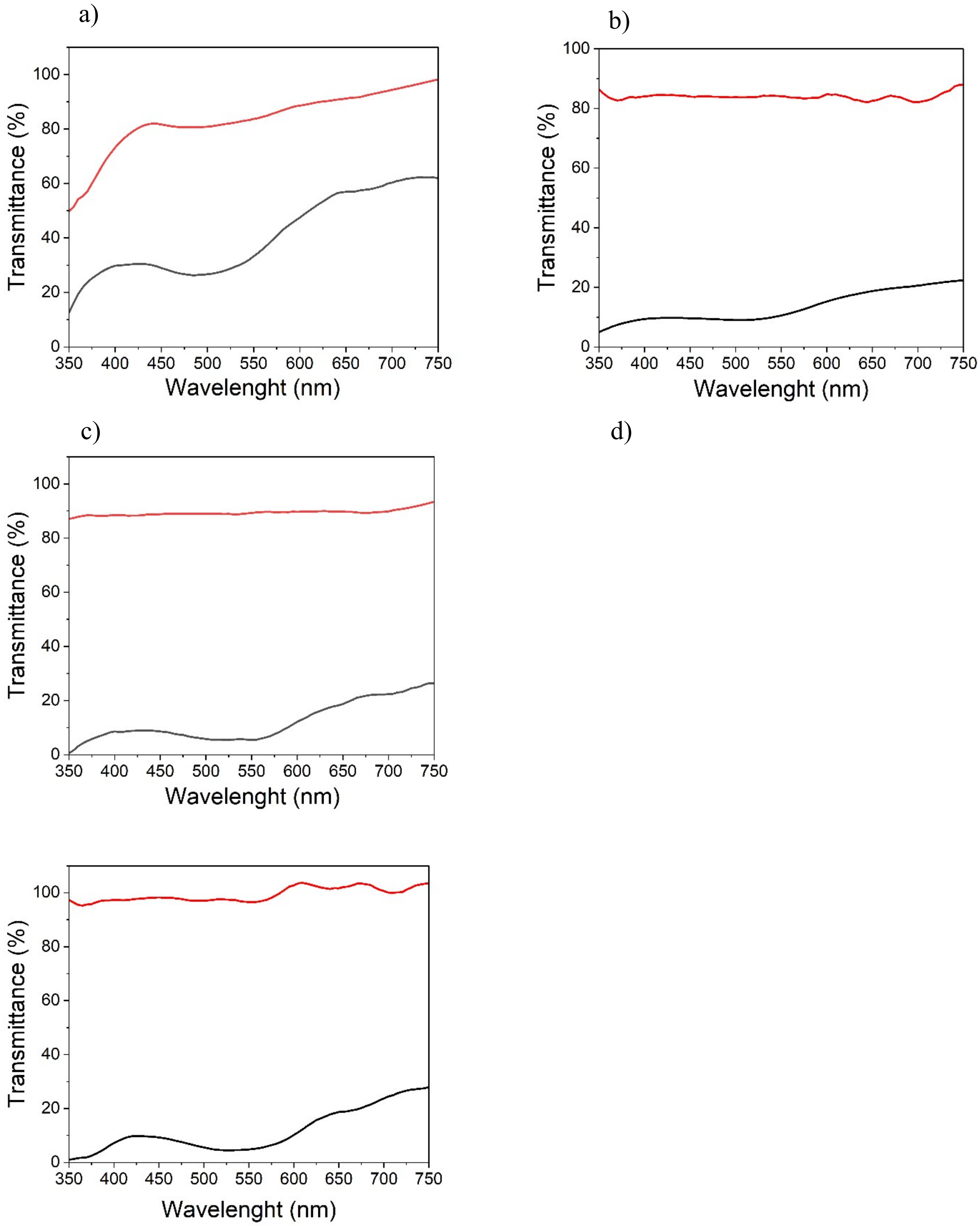

Fig. S5 UV−vis transmittance spectra of (a) RuTPY-UiO-67, (b) Ru2S, (c) Ru3S and (d) Ru4S MOF thin films at colored (black line) and bleached (red line) states.

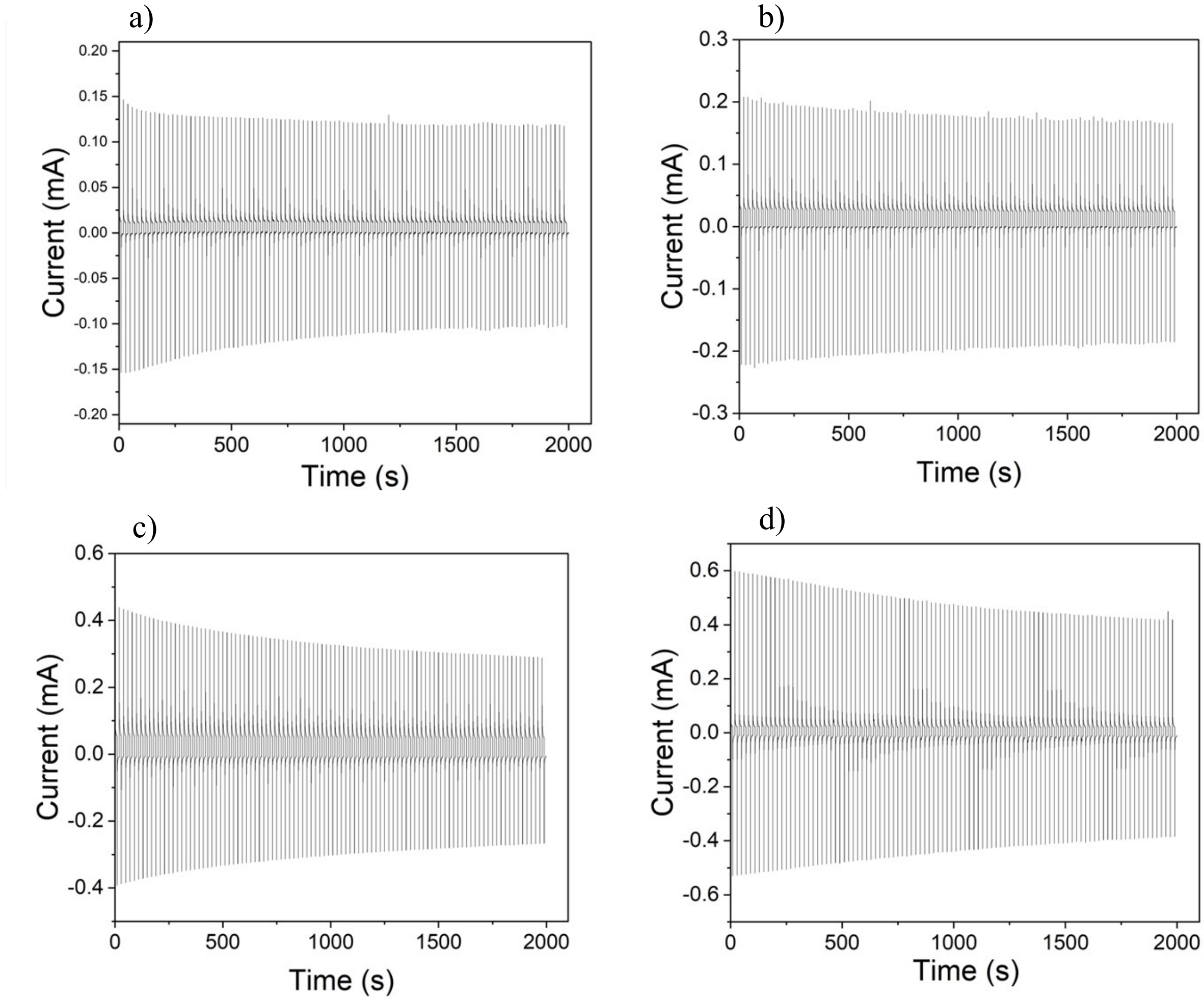

Fig. S6 Stability for 100 cycles of (a) RuTPY-UiO-67, (b) Ru2S, (c) Ru3S and (d) Ru4S MOF thin films.

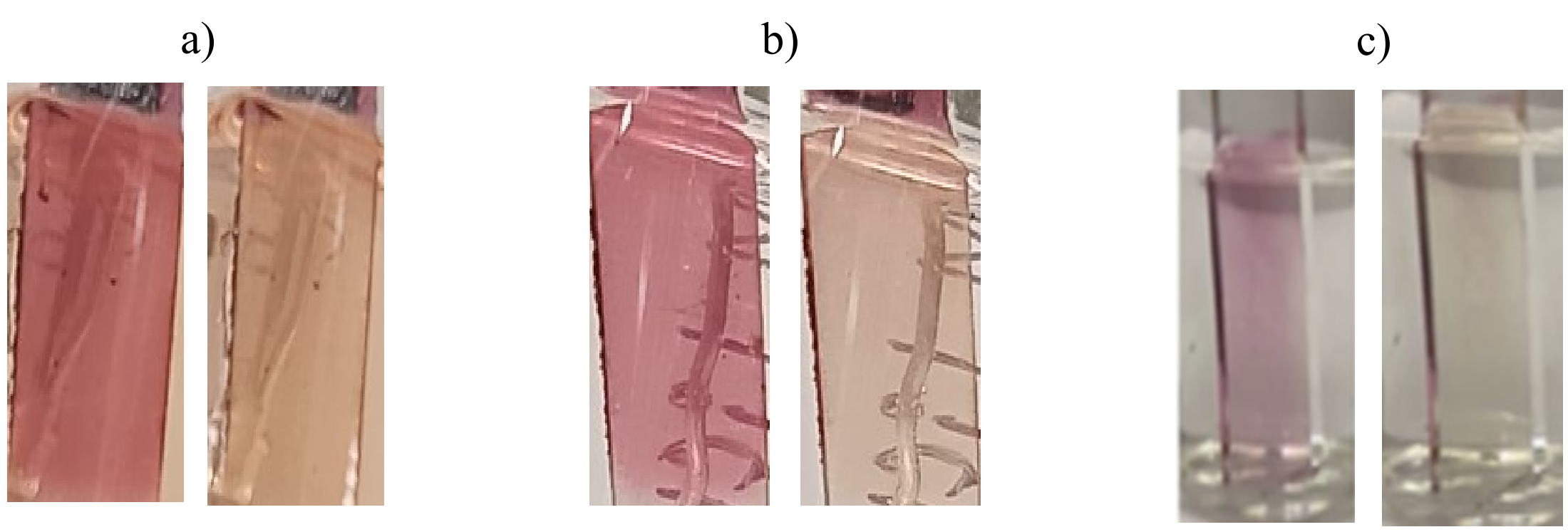

Fig. S7 Photographs of (a) Ru2S, (b) Ru3S and (c) Ru4S MOF thin films in colored (left) and bleached (right) states.

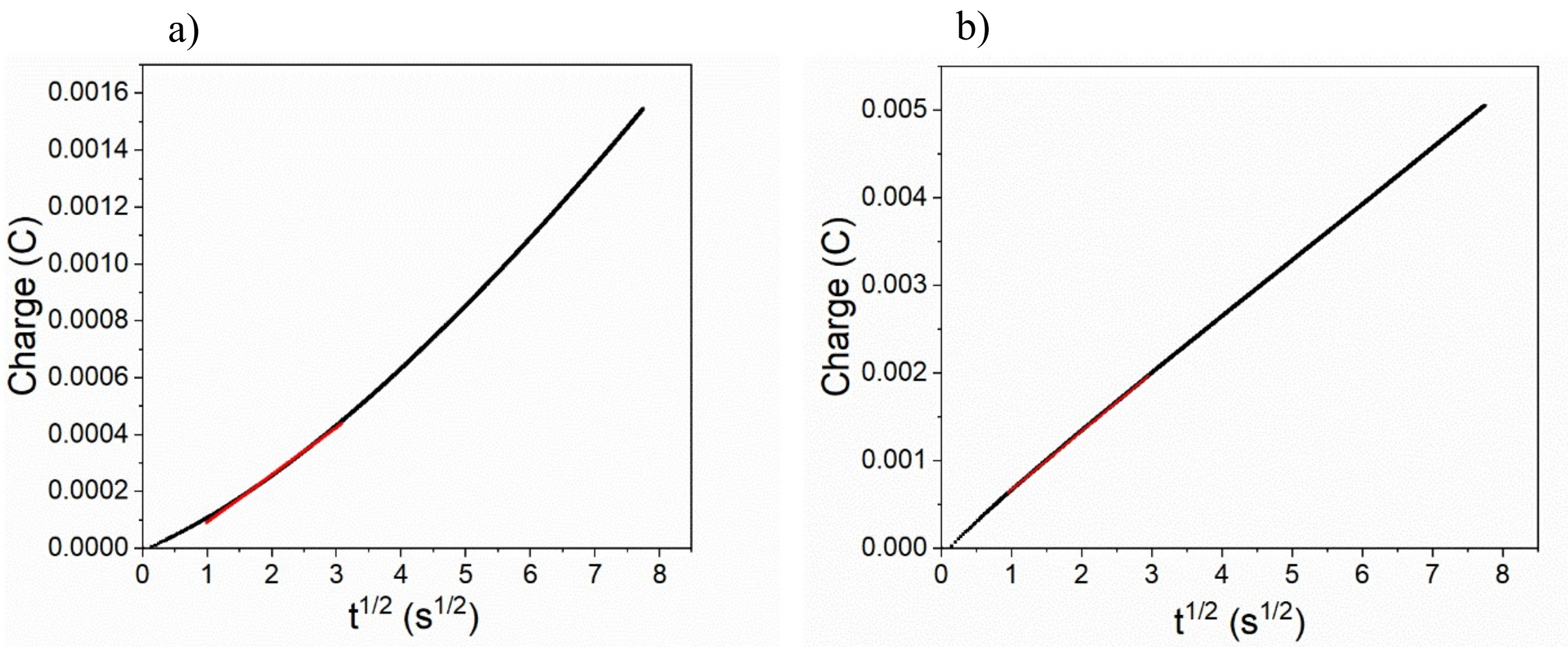

Fig. S8 Chronocoulometric Anson plots for (a) RuTPY-UiO-67 and (b) Ru4S. The red traces highlight the linear regions that were used to extract the slopes for calculating D_{app}s.

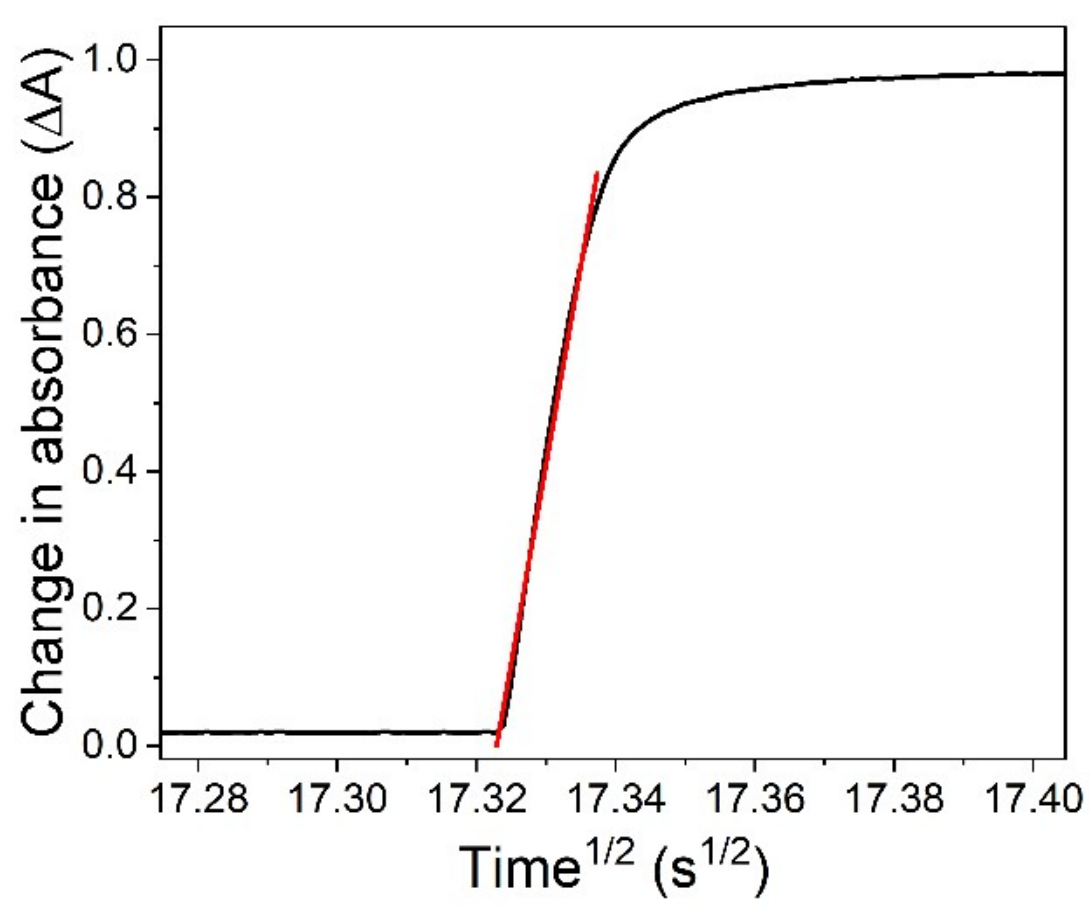

Fig. S9 Plot of ΔA versus $t^{1/2}$ at the characteristic wavelength of the Ru4S MOF thin film. The red line denotes the linear region used to extract the slope, which was then used to calculate the D_{app}.

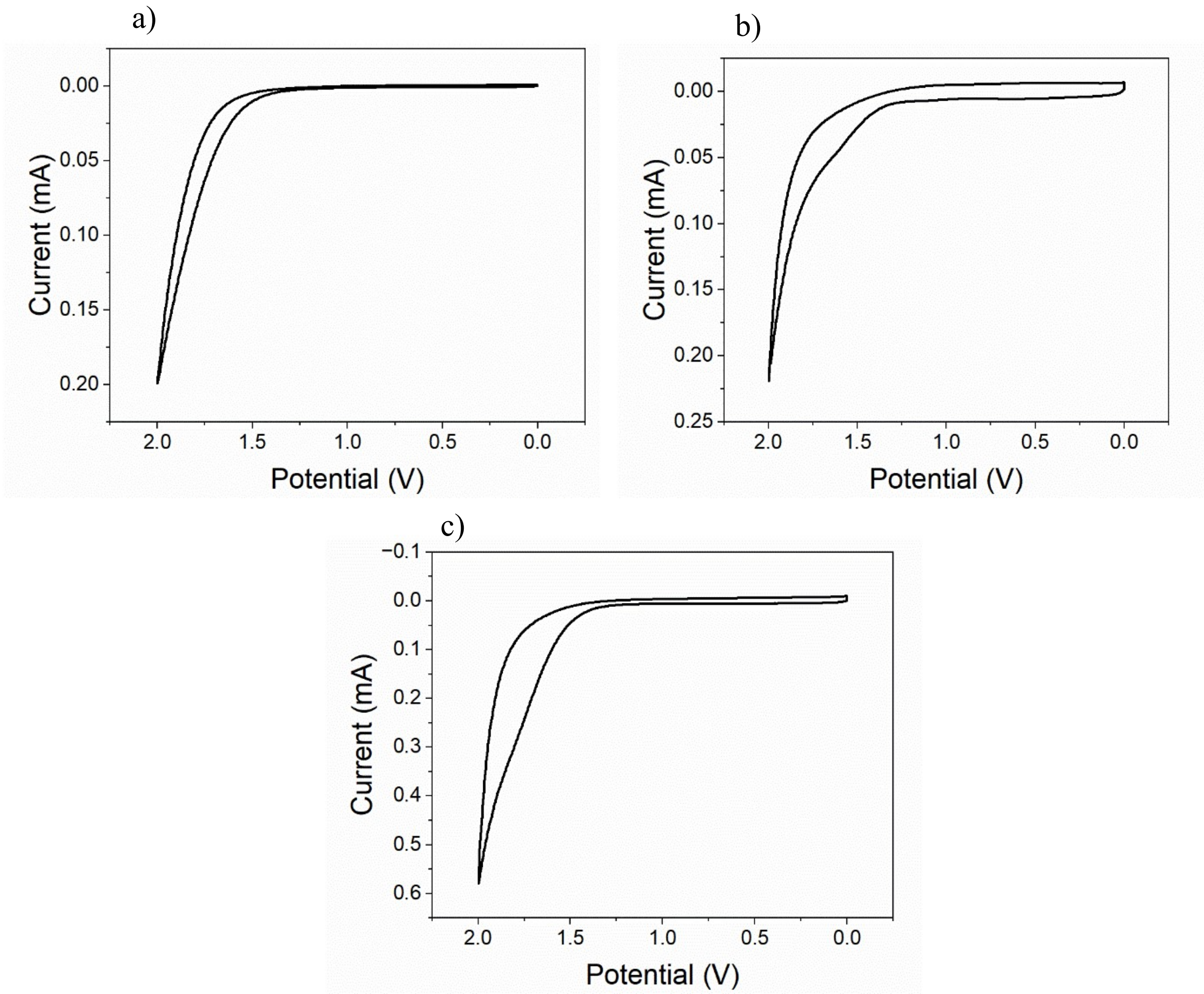

Fig. S10 CVs for chromophore-free (a) Ru2S, (b) Ru3S, and (c) Ru4S. These data prove that the sulfonated frameworks by themself lack redox activity within the investigated potential range.

Table S1. Overview of reported framework-based electrochromic materials in the literature compared with the present study.

Redox-active MOFs	Coloration time (t_c) in seconds	Bleaching time (t_b) in seconds	CE (cm^2/C)	Optical contrast (%)	References
Zn (NDI-ATZ)	4.35	7.72	99.14	6.45	[6]
Zn-TCA (TCA= 4,4',4''-tricarboxytriphenylamine)	4.5	6.2	57	57	[7]
HKUST-1+ZnMOF-74	10	8	N.A.	64	[8]
Cu-HHTP (HHTP = 2,3,6,7,10,11-Hexahydroxytriphenylene)	3.2	5.9	632	40.2	[9]
Ni- BINDI (BINDI = N,N-bis (5-isophthalic acid)-naphthalenediimide)	9.5	5.1	132	42	[10]
Ni-MOF-74	23	29	N.A.	44.4	[11]
Zn-PDI	2.6	1.6	941	96.4	[12]
Zn-NDI	3.7	4	610	97.1	[12]
Zn-PMDI	N.A.	N.A.	753	89.5	[12]
NU-901	5	12	204	64	[13]
$COF_{TAPT\text{-}TFPB}$	18.6	0.7	297.4	50.23	[14]
triphenylamine-based ECOF	13.6	17.8	154.66	25.7	[15]
Imine-linked COF	4.9	10.6	144	~60%	[16]
COF-GZU1	~7.8–14.5	~1.0–12.6	112.8-127	N.A.	[17]
Py-ttTII COF	0.2	0.4	858	N.A.	[18]

Ru2S	4.25	5.26	225	57.1	This Work
Ru3S	2.65	3.45	351	78.2	This Work
Ru4S	1.09	1.5	641	89.2	This Work

Table S2. Overview of reported diffusion coefficients of MOFs in the literature compared with the present study.

**Redox-active MOFs**	**Diffusion coefficient (cm^2/s)**	**References**
Metallocene-doped NU-1000	$\sim 10^{-10} – 10^{-9}$	[19]
CoTCPP (TCPP = [5,10,15,20-(4-carboxyphenyl)porphyrin])	$7.55(\pm 0.05)\times 10^{-14}$	[20]
NU-1000	2×10^{-10}	[21]
Zr-(dcphOH-NDI)	$5.4(\pm 1.1)\times 10^{-11}$	[22]
Ir-UiO-66	10^{-12}	[23]
Hemin-UiO-66	10^{-9}	[24]
Ru2S	$(3 \pm 2) \times 10^{-8}$	This Work
Ru3S	$(1.3 \pm 0.5) \times 10^{-7}$	This Work

Ru4S	$(5 \pm 3) \times 10^{-6}$	This Work

Table S3. Overview of reported MOF cycling stability in the literature compared with the present study.

Electrochromic MOFs	Supporting electrolyte	Substrate	Cycle number	References
Zn(NDI-H) [NDI = naphthalenediimide]	0.1 M $[(nBu)_4N]PF_6$/DMF	FTO	25	25
Ni-NDISA	0.1 M $[(nBu)_4N]PF_6$/DMF	FTO	10	26
Zn-DSNDI	0.1 M $[(nBu)_4N]PF_6$/DMF	ZnO/FTO	10	27
Zr-BINDI [BINDI = N,N′-bis (5-isophthalic acid) naphthalenediimide]	0.1 M $[(nBu)_4N]PF_6$/DMF	ITO	100	28
Zn(NDIATZ)(NBU-3)	0.1 M $[(nBu)_4N]PF_6$	FTO	6	29
N-doped NiO@C Ni MOF	1 M KOH solution	ITO	50	30
NU-901 (Zr-TBAPy)	0.1 M $TBAPF_6$/ DCM	FTO	60	31
Mg-MOF-74	0.2 M $LiClO_4$/ MeCN	FTO	100	32
Zn-NDI	0.5 M KPF_6/ DMF	FTO	50	12
Ni-HITP205 [HITP = Hexaiminotriphenylene]	0.1 M $LiClO_4$/PC (PC = Propylene carbonate)	FTO	100	33
Cu-TCA [TCA=tricarboxytriphenylamine]	0.1 M $LiClO_4$/PC	ITO	500	34
$Cu_3(HHTP)_2$ [HHTP = 2,3,6,7,10,11-hexahydroxytriphenylene]	0.1 M $LiClO_4$/PC	FTO	100	35
Ru2S	0.1 M $LiClO_4/CH_3CN$	FTO	100	This Work

Ru3S	0.1 M $LiClO_4/CH_3CN$	FTO	100	This Work
Ru4S	0.1 M $LiClO_4/CH_3CN$	FTO	100	This Work

References

1 B. Gibbons, D. R. Cairnie, B. Thomas, X. Yang, S. Ilic and A. J. Morris, *Chem. Sci.*, 2023, **14**, 4672–4680.
2 J. Zhao, X. He, Y. Zhang, J. Zhu, X. Shen and D. Zhu, *Cryst. Growth Des.*, 2017, **17**, 5524–5532.
3 L.-J. Zhou, W.-H. Deng, Y.-L. Wang, G. Xu, S.-G. Yin and Q.-Y. Liu, *Inorg. Chem.*, 2016, **55**, 6271–6277.
4 4 Q. Chen, F. Shao, G. Wu, D Izuhara and H. Umeda, *WO 2013/040985 A1*, 2016.
5 S. Lin, Y. Pineda-Galvan, W. A. Maza, C. C. Epley, J. Zhu, M. C. Kessinger, Y. Pushkar and A. J. Morris, *ChemSusChem*, 2017, **10**, 514–522.
6 P. P. More, P. V. Rathod, J. M. C. Puguan and H. Kim, *Dyes Pigm.*, 2021, **195**, 109730.
7 J. Liu, X. Y. D. Ma, Z. Wang, L. Xu, F. Wang, C. He and X. Lu, *ACS Appl. Electron. Mater.*, 2021, **3**, 1489–1495.
8 I. Mjejri, C. M. Doherty, M. Rubio-Martinez, G. L. Drisko and A. Rougier, *ACS Appl. Mater. Interfaces*, 2017, **9**, 39930–39934.
9 R. Li, S. Li, Q. Zhang, Y. Li and H. Wang, *Inorg. Chem. Commun.*, 2021, **123**, 108354.
10 R. Li, K. Li, G. Wang, L. Li, Q. Zhang, J. Yan, Y. Chen, Q. Zhang, C. Hou, Y. Li and H. Wang, *ACS Nano*, 2018, **12**, 3759–3768.
11 N. Zhang, Y. Jin, Q. Zhang, J. Liu, Y. Zhang and H. Wang, *Ionics*, 2021, **27**, 3655–3662.
12 A. Kumar, J. Li, A. K. Inge and S. Ott, *ACS Nano*, 2023, **17**, 21595–21603.
13 C.-W. Kung, T. C. Wang, J. E. Mondloch, D. Fairen-Jimenez, D. M. Gardner, W. Bury, J. M. Klingsporn, J. C. Barnes, R. Van Duyne, J. F. Stoddart, M. R. Wasielewski, O. K. Farha and J. T. Hupp, *Chem. Mater.*, 2013, **25**, 5012–5017.
14 R.-Z. Li, Q. Hao, X.-R. Ren, C. Wen, L. Wang, Z.-L. Zhao, J.-Y. Shao, Y.-W. Zhong, D. Wang and L.-J. Wan, *ACS Appl. Mater. Interfaces*, 2024, **16**, 49594–49601.
15 Q. Chen, Y. Wang, J. Yu, Y. Si and P. Tang, *ACS Appl. Mater. Interfaces*, 2025, **17**, 67092–67102.
16 T. K. Dutta, S. Das, M. Sarkar, M. Bhattacharjee and A. Patra, *Chem. Mater.*, 2024, **16**, 8027–8036.
17 Y. Wang, R. Zheng, H. Li, Y. Liu, M. Tian, W. Wu, Q. Liu and C. Jia, *Chem. Eng. J.*, 2024, **497**, 154818.
18 D. Bessinger, K. Muggli, M. Beetz, F. Auras and T. Bein, *J. Am. Chem. Soc.*, 2021, **143**, 7351–7357.
19 P. J. Celis-Salazar, M. Cai, C. A. Cucinell, S. R. Ahrenholtz, C. C. Epley, P. M. Usov and A. J. Morris, *J. Am. Chem. Soc.*, 2019, **141**, 11947–11953.
20 S. R. Ahrenholtz, C. C. Epley and A. J. Morris, *J. Am. Chem. Soc.*, 2014, **136**, 2464–2472.
21 S. Goswami, I. Hod, J. D. Duan, C.-W. Kung, M. Rimoldi, C. D. Malliakas, R. H. Palmer, O. K. Farha and J. T. Hupp, *J. Am. Chem. Soc.*, 2019, **141**, 17696–17702.

22 B. A. Johnson, A. Bhunia, H. Fei, S. M. Cohen and S. Ott, *J. Am. Chem. Soc.*, 2018, **140**, 2985–2994.

23 C.-H. Chuang, J.-H. Li, Y.-C. Chen, Y.-S. Wang and C.-W. Kung, *J. Phys. Chem. C*, 2020, **124**, 20854–20863.

24 R. Shimoni, W. He, I. Liberman and I. Hod, *J. Phys. Chem. C*, 2019, **123**, 5531–5539.

25 C. R. Wade, M. Li and M. Dincă, *Angew. Chem. Int. Ed.*, 2013, **52**, 13377–13381.

26 K. AlKaabi, C. R. Wade and M. Dincă, *Chem*, 2016, **1**, 264–272.

27 X. Wu, K. Wang, J. Lin, D. Yan, Z. Guo and H. Zhan, *J. Colloid Interface Sci.*, 2021, **594**, 73–79.

28 G. Radha, S. Roy, C. Chakraborty and H. Aggarwal, *Chem. Commun.*, 2022, **58**, 4024–4027.

29 N. Zhang, Y. Jin, Q. Zhang, J. Liu, Y. Zhang and H. Wang, *Ionics*, 2021, **27**, 3655–3662.

30 Z. Zeng, X. Peng, J. Zheng and C. Xu, *ACS Appl. Mater. Interfaces*, 2021, **13**, 4133–4145.

31 C.-W. Kung, T. C. Wang, J. E. Mondloch, D. Fairen-Jimenez, D. M. Gardner, W. Bury, J. M. Klingsporn, J. C. Barnes, R. Van Duyne, J. F. Stoddart, M. R. Wasielewski, O. K. Farha and J. T. Hupp, *Chem. Mater.*, 2013, **25**, 5012–5017.

32 H. Shiozawa, Z. Melnikova, Z. Bastl, H. Peterlik, M. Kalbac and O. Frank, *Adv. Photon. Res.*, 2022, **3**, 2100219.

33 L. Pan, R. Li, C. Zhang, Z. Lu, K. Li, Q. Zhang, C. Hou, Y. Li and H. Wang, *ACS Appl. Electron. Mater.*, 2022, **4**, 2915–2922.

34 S. Feng, J. Wang, Z. Tong and H.-Y. Qu, *Chem. Eng. J.*, 2022, **442**, 136158.

35 R. Li, S. Li, Q. Zhang, Y. Li and H. Wang, *Inorg. Chem. Commun.*, 2021, **123**, 108354.
